# A novel miR-1291-ERRα-CPT1C axis modulates tumor cell proliferation, metabolism and tumorigenesis

**DOI:** 10.7150/thno.44877

**Published:** 2020-06-01

**Authors:** Yixin Chen, Yanying Zhou, Fangwei Han, Yingyuan Zhao, Meijuan Tu, Yongtao Wang, Can Huang, Shicheng Fan, Panpan Chen, Xinpeng Yao, Lihuan Guan, Ai-Ming Yu, Frank J. Gonzalez, Min Huang, Huichang Bi

**Affiliations:** 1 School of Pharmaceutical Sciences, Sun Yat-sen University, Guangzhou, China 510006.; 2 School of Public Health, UNT Health Science Center, Fort Worth, TX 76107, USA.; 3 Department of Biochemistry & Molecular Medicine, UC Davis School of Medicine, Sacramento, CA 95817, USA.; 4 Laboratory of Metabolism, Center for Cancer Research, National Cancer Institute, NIH, Bethesda, MD 20892, USA.

**Keywords:** miR-1291, estrogen-related receptor α, carnitine palmitoyltransferase 1C, cell proliferation, tumor metabolism

## Abstract

**Rationale:** MicroRNAs are known to influence the development of a variety of cancers. Previous studies revealed that miR-1291 has antiproliferative functions in cancer cells. Carnitine palmitoyltransferase 1C (CPT1C) has a vital role in mitochondrial energy metabolism and modulation of cancer cell proliferation. Since both miR-1291 and CPT1C regulate tumor cell metabolism and cancer progression, we hypothesized that they might be regulated synergistically.

**Methods:** A series of cell phenotype indicators, such as BrdU, colony formation, cell cycle, ATP production, ROS accumulation and cell ability to resist metabolic stress, were performed to clarify the effects of miR-1291 and ERRα expression on tumor cell proliferation and metabolism. A xenograft tumor model was used to evaluate cell tumorigenesis. Meta-analysis and bioinformatic prediction were applied in the search for the bridge-link between miR-1291 and CPT1C. RT-qPCR, western-blot and IHC analysis were used for the detection of mRNA and protein expression. Luciferase assays and ChIP assays were conducted for in-depth mechanism studies.

**Results:** The expression of miR-1291 inhibited growth and tumorigenesis as a result of modulation of metabolism. CPT1C expression was indirectly and negatively correlated with miR-1291 levels. *ESRRA* was identified as a prominent differentially expressed gene in both breast and pancreatic cancer samples, and estrogen-related receptor α (ERRα) was found to link miR-1291 and CPT1C. MiR-1291 targeted ERRα and CPT1C was identified as a newly described ERRα target gene. Moreover, ERRα was found to influence cancer cell metabolism and proliferation, consistent with the cellular changes caused by miR-1291.

**Conclusion:** This study demonstrated the existence and mechanism of action of a novel miR-1291-ERRα-CPT1C cancer metabolism axis that may provide new insights and strategies for the development of miRNA-based therapies for malignant cancers.

## Introduction

MicroRNA-1291 (miR-1291) is generated from SNORA34 (small nucleolar RNA H/ACA box 34) in the pancreatic cancer cell line PANC-1 1. In previous studies, miR-1291 was reported to have a variety of functions. Expression of miR-1291 was significantly reduced in clinical samples of pancreatic cancer 2. miR-1291 was shown to modulate cellular drug chemosensitivity and disposition by targeting multidrug resistance-associated protein 1 (MRP1/ABCC1) 1,3 . Moreover, miR-1291 directly affects a variety of metabolic pathways, such as fork head box protein A2 (FOXA2) and glucose transporter 1 (GLUT1), to affect the growth and invasion of tumor cells 4,5. However, the molecular mechanisms underlying these important functions have not to be elucidated.

Another biomarker of cancer metabolism is CPT1C, a member of the CPT1 family that catalyzes fatty acid acylation and entry into mitochondria for β-oxidation 6. CPT1C is involved in the regulation of energy homeostasis, ceramide metabolism and the control of food intake by the hypothalamus 7-9. Recently, it was revealed that CPT1C levels are involved in the poor prognosis and metastatic progression of human cancers, which are closely related to fatty acid uptake and metabolism 10. Most recently, CPT1C was found to be a critical regulator of tumor cell proliferation and senescence through mitochondria-associated metabolic reprogramming, and is a potential novel target that controls tumor progression 11.

Since both miR-1291 and CPT1C were identified as tumor biomarkers in pancreatic cancer 1,2,11 and both are closely related to tumor metabolism 4,5,12, the assumption was made that they may be mechanistically linked in their impact on cancer. Furthermore, through Meta-analysis and bioinformatic prediction, ERRα was found in the present study to be the conduit between miR-1291 and CPT1C.

ERRα is encoded by the *ESRRA* gene and is an orphan member of the nuclear receptor superfamily. As a transcription factor, ERRα mediates mitochondrial biogenesis and also operates as a master regulator of cellular energy metabolism by regulating genes involved in fatty acid metabolism, the tricarboxylic acid cycle or oxidative phosphorylation 13,14. In addition to the normal metabolism, ERRα shows more noticeable functions in various malignancies 15-17. The occurrence and prognosis of a wide range of carcinomas, such as breast cancer, prostate cancer, colorectal cancer and ovarian cancer, were reported to be associated with ERRα as well as the ERRα/PGC1α complex 16,18,19.

Therefore, the objective of the current study was to dissect the regulatory mechanism of the miR-1291-ERRα-CPT1C axis and to explain how each synergistically works on tumor cell metabolism and proliferation. Here, the explicit action of miR-1291 on tumors was explored via the ERRα-CPT1C pathway. Both CPT1C and upstream ERRα account for the antineoplastic potential of miR-1291. Investigation of miRNA regulatory pathways will provide insights into the identification of novel oncotargets and the development of new cancer therapeutic agents 20,21.

## Materials and Methods

### Cell culture

The human pancreatic cancer cell line PANC-1 was purchased from Guangzhou Cellcook Biotech Company. The human breast cancer cell line MDA-MB-231 and the embryonic kidney 293T cell line were provided by Dr. Jun Du at Sun Yat-sen University. The cells were maintained in Dulbecco's modified Eagle's medium (Corning, USA) with 4.5 g/L glucose, L-glutamine and sodium pyruvate supplemented with 10% FBS (Gibco, USA), 1% streptomycin sulfate and penicillin sodium (Gibco, USA) at 37 °C in a humidified atmosphere of 5% CO_2_. These cell lines were authenticated every year by the Guangzhou Cellcook Biotech Company using Short Tandem Repeat Authentication. Cells were monitored for mycoplasma contamination using Myco-Lumi Mycoplasma Detection Kit (Beyotime Biotech, China). PANC-1 and MDA-MB-231 cells stably transfected with miR-1291 were named ST-miR1291-PANC-1 or ST-miR1291-231, respectively, and were established recently by Wuhan Gene Create Company, China. The control cell lines named Control-PANC-1 or Control-231 with the same pCDH-CMV-MCS-EF1-GFP-Pruo empty vectors were developed in the same manner.

### Transfection of plasmids and siRNA

The coding sequence of the ERRα (ESRRA) mRNA-3'UTR segment consisting of miR-1291 MRE (miRNA response elements) sites was predicted by TargetScan database (http://www.targetscan.org/). The miR-1291 expression plasmid and a series of *ESRRA* 3'UTR reporter plasmids were constructed by Wuhan Gene Create Company. Human ERRα DNA was subcloned into the pENTER vector (Vigene, China). The accuracy of plasmids was confirmed by DNA sequencing. The miR-1291 and ERRα overexpression plasmids vectors were transfected at a concentration of 1 µg/10^6^ cells using Mega DNA Transfection Reagent (Origene, USA) with the reduced serum medium Opti-MEM (Gibco, USA). For specific RNA interference and miRNA inhibition experiments, small interfering RNAs (siRNA) or high affinity miRNA inhibitor (Ribobio, China) were used to decrease ERRα, CPT1C or miR-1291 levels. Cells were transfected with 50 nM siRNA or 100 nM miRNA inhibitor using Lipofectamine RNAiMAX Transfection Reagent (Invitrogen, USA) with Opti-MEM (Gibco, USA). The effectiveness of these different siRNA chains, plasmid and inhibitor were determined through RT-qPCR analysis, and the most effective siRNA chain was chosen for all experiments. The methods of all transfections can be found in the manufacturer's protocols.

### WST-8 assay and BrdU assay

The viability and proliferation capacity of tumor cells were analyzed by water-soluble tetrazolium-8 (WST-8) and bromodeoxyuridine (BrdU) assays, respectively. The cells were seeded into 96-well plates. Forty eight hours after transfection, WST-8 activity was measured by the addition of 10 μL of WST reagent (Beyotime Biotech, China) to each well. After incubating for 1-3 h at 37 °C, the absorbance was measured at a wavelength of 450 nm. BrdU incorporation was measured using a Cell Proliferation ELISA, BrdU (colorimetric) Kit (Roche, Switzerland). In brief, 10 μL BrdU labeling solution was added to the cells in each well and was then incubated for 2 h. Anti-BrdU-POD working solution served as one specific antibody, and the absorbance was read at a 370 nm wavelength.

### Colony formation and cell cycle analysis

Colony formation assays were performed by seeding 2,500 cells per well in 6-well plates after transfection for 48 h and allowing the cells to form colonies for 14 days. The cells were fixed with formaldehyde and stained with Diff-Quik (Propbs, China) reagent for 3 min. For cell cycle analysis, the cells were washed twice with PBS and were fixed with 70% cold ethanol at 4 °C overnight. The cells were then centrifuged and resuspended in PBS and were then stained with 0.5 mL propidium iodide for 30 min at 37 °C in the dark according to the protocol of the Cell Cycle and Apoptosis Analysis Kit (Beyotime Biotech, China). The data were acquired using flow cytometry (Beckman Coulter, USA) at a 488 nm wavelength, and processed with FlowJo Version 7.6.1.

### ATP and ROS production analysis

For the total ATP production analysis, the culture medium in 96-well plates was replaced with PBS for 12 h. The luminescence intensity represented the cellular ATP levels and was adjusted to the amount of the total protein levels. The intensity was measured with the CellTiter-Glo Luminescent Cell Viability Assay (Promega, USA) in Flex Station 3 (Molecular Devices, USA). To estimate the intracellular ROS levels, the ROS accumulation was examined with a Reactive Oxygen Species Assay Kit (Beyotime Biotech, China). After washing with PBS, the cells were pretreated with 10 mM DCFH-DA for 20 min and then detected with a High Content Screening System ArrayScanVTI (Thermo, USA).

### Metabolic stress stimuli analysis

To explore the anti-metabolic stress ability of tumor cells, an analysis of metabolic stress stimuli was performed as follows: 2-Deoxyglucose (2-DG) (Sigma, USA) was dissolved in DMSO and was diluted serially to the required concentrations in culture medium. For the glucose deprivation test, the cells were incubated in a glucose-deficient medium which was supplemented with a stock glucose solution (Sigma, USA) to the indicated concentrations. The cells were further subjected to preoptimized 2-DG or glucose. After 72 h of culture, the cell growth was measured by WST-8 assay.

### Xenograft tumor study

To show the effects of miR-1291 on cellular tumorigenicity, mouse xenograft assays were performed. Athymic male nude 4- to 5-week-old BALB/c-nu/nu mice were purchased from the Experimental Animal Center of Sun Yat-sen University and maintained under a standard 24 h cycle (12 h light/12 h dark) with chow and water provided *ad libitum*. A total of 5×10^6^ tumor cells that were growing exponentially were harvested and injected subcutaneously in the right flank of each mouse with 50% Matrigel basement membrane matrix (BD, USA). The tumor sizes and body weights were monitored twice a week for 3 to 4 weeks. The mice were killed when the tumor sizes exceeded 1.5 cm in diameter, and the xenograft tumor samples harvested. All animal studies were approved by the Institutional Animal Care and Use Committee of Sun Yat-sen University.

### Quantitative real-time PCR analysis

Total RNA from cultured cells was extracted using Trizol Reagent (Invitrogen, USA), and the miRNA isolated using a miRcute miRNA Kit (TIANGEN, China). Single-stranded complementary DNA was synthesized with a reverse transcription reaction using a Primer Script RT Reagent Kit (TaKaRa, Japan) or miRcute miRNA-cDNA Kit (TIANGEN, China). All sequences of primers used for quantitative RT-PCR are shown in Tables S1 and S2. The mRNAs and miRNAs were then amplified via a Biosystems 7500 Real-time PCR system using SYBR Premix Ex-Taq II Kit (TaKaRa, Japan) or miRcute miRNA qPCR Detection Kit (TIANGEN, China) according to the manufacturer's protocols. The fold changes were analyzed using the ΔΔCt method.

### Western blot analysis

Total proteins from cultured cells were lysed using RIPA lysis buffer containing 1% 100 mM phenylmethanesulfonyl fluoride and were then quantified using a BCA Protein Assay Kit (Thermo, USA). The protein expression levels were analyzed by western blotting. Forty micrograms of protein per lane was separated on a 10% SDS-PAGE gel and transferred to polyvinylidene fluoride membranes. After blocking, the blots were incubated overnight at 4 °C with different antibodies against GAPDH (Santa Cruz Biotechnology, USA, Cat# sc-25778), ERRα (Cell Signaling Technology, USA, Cat# 13826) , cyclin D1 (Cell Signaling Technology, USA, Cat# 2978), cyclin A1 (Sangon Biotech, China, D151774), cyclin E1 (Cell Signaling Technology, USA, Cat# 20808), and CPT1C (Abcam, USA, Cat# ab123794) followed by an incubation with the secondary anti-rabbit (Cell Signaling Technology, USA, Cat# 7074) or anti-mouse antibodies (Cell Signaling Technology, USA, Cat# 7076) at room temperature on the following day. The ECL Detection Kit (Engreen Biosystem, China) was applied to develop the blots. The intensity of the protein bands was assayed by Quantity One software (Quantity One 1-D Analysis Software).

### Immunohistochemistry analysis

MDA-MB-231 cells were fixed with 4% paraformaldehyde in PBS for 30 minutes, permeabilized with 0.2% Triton X-100 for 10 minutes and incubated in a 10% serum blocking solution for 1 h at room temperature. Primary ERRα (Santa Cruz Biotechnology, USA) or CPT1C (Abcam, USA) antibodies were diluted in serum blocking solution and were incubated overnight at 4 °C. HRP-labeled anti-rabbit or anti-mouse secondary antibodies were applied respectively for 1 h. ICC was developed using the DAB+ Chromogen System (Dako, USA), and the nuclei counterstained with hematoxylin.

### Luciferase activity assay

HEK-293T cells plated in 96-well plates were co-transfected with the following plasmids: an *ERRα*-Reporter-WT luciferase reporter plasmid containing the *ESRRA* 3'UTR region, a miR-1291 expression plasmid or a vector-control plasmid, and a plasmid luc-TK. We also performed a luciferase assay on the mutant binding sites of *ESRRA* 3'UTR using *ERRα*-Reporter-MUT plasmids.

The luciferase assay of ERRα expression to *CPT1C* promoters was performed as described previously 22. Cells were transfected together with a series of plasmids including CPT1C reporter plasmids, which were inserted with different lengths of the *CPT1C* promoter regions, the expression plasmid pENTER-ERRα as well as plasmid luc-TK. CPT1C reporter plasmids, in which certain ERRE sequences were mutated, were also subjected to luciferase assays to elucidate the important types and numbers of binding sites for the transcriptional activation. The cells were incubated for 24 h prior to lysis with Passive Lysis Buffer. A luciferase enzymatic activity was determined by a commercial Dual-Luciferase Reporter Assay System Kit (Promega, USA).

### Chromatin immunoprecipitation (ChIP)-PCR assay

Chromatin immunoprecipitation assays were performed by using a Pierce Agarose ChIP Kit (Thermo, USA). Specifically, four segments containing several potential ERREs (ERR responsive element) were first divided from the entire CPT1C promoter according to the positions of the binding sites. The genomic DNA was crosslinked to nuclear proteins by formaldehyde. Subsequently, the cells were collected in cold PBS containing protease inhibitors. Cross-linked chromatin complex was digested by an optimized enzymolysis method. Most DNA fragments range in length from 100 to 500 bp, which would allow for further experiments. Following centrifugation, the pellets were resuspended in lysis buffer. At 4 °C, the total chromatin DNA fragments were precipitated using an anti-ERRα antibody (Cell Signaling Technology, USA) overnight. After adding protein G-sepharose beads, the resultant immune complexes were washed sequentially with wash buffer. Then, the immune complexes were suspended again in elution buffer. The primers used for ChIP-PCR were designed based on the target fragment sequences, which are shown in Table S3. Human genomic DNA was amplified by real-time qPCR according to the putative ERRE binding sites identified *in silico*. The input sample lane and IgG antibody served as the positive and negative controls, respectively.

### Meta-analysis

Oriented by the goal of finding the key factor that link miRNA-1291 and CPT1C, also working with them to regulate cancer progression, a meta-analysis of multiple types of studies was performed. We generated queries that mined all Series (GSE) related to breast cancer and pancreatic cancer from Gene Expression Omnibus (GEO) database. The next step was to manually select series that met certain criteria (ie. Has to have at least 3 tumor samples and 3 normal samples; has to use platforms of Affymetrix HG-U133 or higher.) Table S4 shows the selected Microarray GSE data summarization. A linear model was fitted to each dataset to calculate univariate Fold Change (FC) and p-value for each mapped gene using limma 23. Then, weighted FC and Fisher's method of combining p-values were used to combine the statistical results from each dataset according to Fisher, R. A.: Statistical Methods for Research Workers, 4th Edition. Specifically, the weight of FC was calculated as the reciprocal of the variation of the corresponding gene expression. All gene candidates were selected with logFC greater than 0 and p-value less than 0.05. The gene candidates derived from meta-analysis can be described as genes that are significantly upregulated in tumor samples, comparing with normal sample (Table S5, sheet1 shows upregulated gene list of pancreatic tumor, sheet2 shows upregulated gene list of breast tumor). To extract the real connection from these selected genes, certain criteria must be met: the gene must be potential miR-1291 target (Table S6 shows the list of potential miR-1291 targets according to Targetscan Database) and should be a transcription factor (Table S7 shows Transcription Tactors list according to JASPAR Database). Therefore, an intersection was generated to refine the results. The workflow of the Meta-analysis was showed in Figure S1.

### Statistical Analysis

All data were presented as the mean ± standard deviation (mean ± SD). Two-tailed Student's *t-*tests were used to assess the differences between the groups using SPSS 19.0 software and GraphPad Prism 7.0 software. Each experiment was independently repeated at least three times. The significance was represented by * *p* < 0.05, **p < 0.01, ***p < 0.001 versus the vector or control groups.

## Results

### miR-1291 inhibits cancer cell proliferation and metabolism

**Figure 1:**
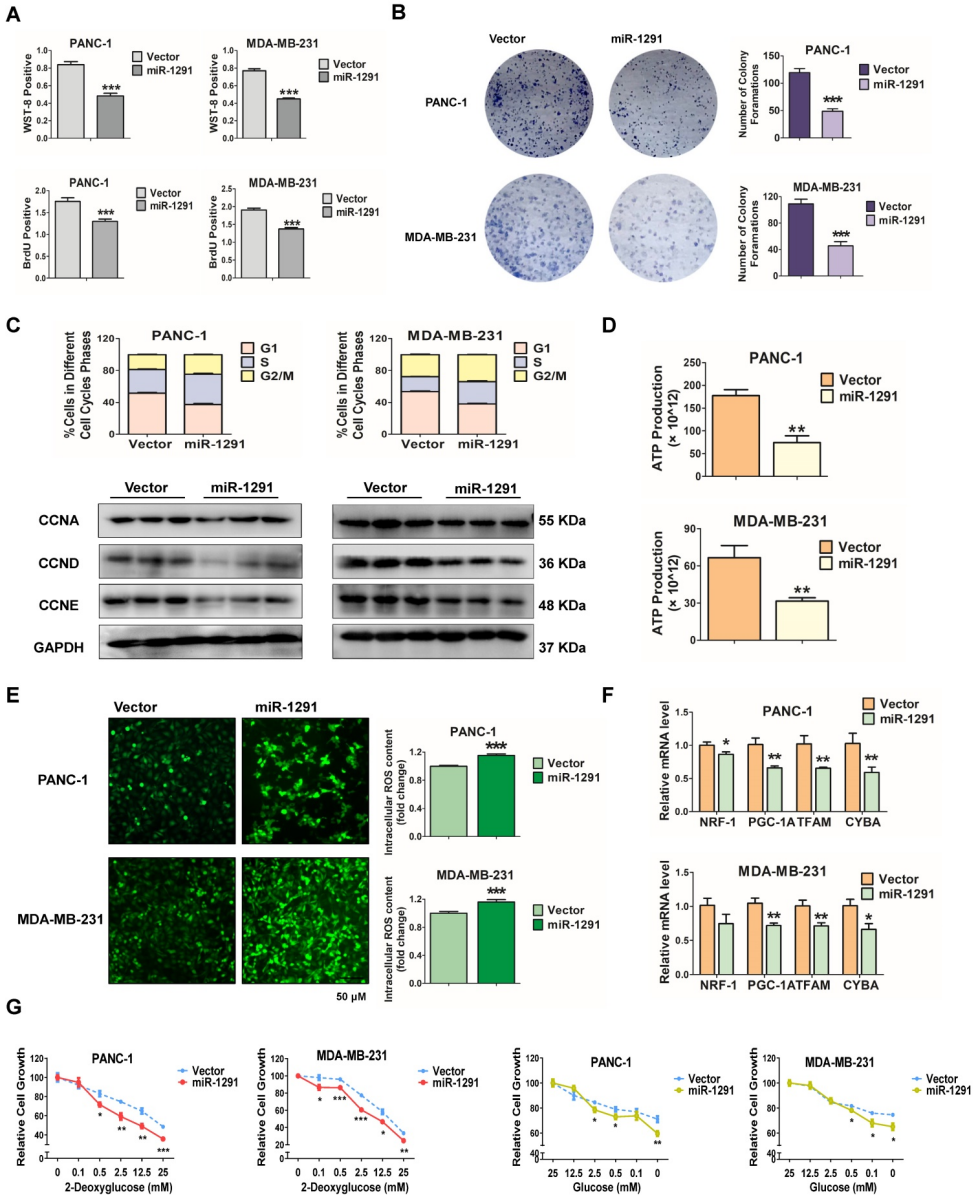
** miR-1291 inhibits cancer cell proliferation and metabolism. (A)** A WST-8 assay was performed to examine the viability of PANC-1 and MDA-MB-231 cells after overexpression of miR-1291. BrdU activity was used to measure cell proliferation capacity after treated with miR-1291 plasmid. Data are mean ± SD (*n* = 5). **(B)** For colony formation assays, the cells were stained with Diff-Quik after being cultured for an additional 14 days. **(C)** The cell cycle was determined by flow cytometry after transfection with miR-1291 and immunoblot analysis of cell cycle-related proteins, such as cyclin A/D/E after transfection with miR-1291. Data are mean ± SD (*n* = 3). **(D)** ATP production in miR-1291-transfected cells. Data are mean ± SD (*n* = 5). **(E)** Intracellular accumulation of ROS in two cell lines. Data are mean ± SD (*n* = 5). **(F)** RT-qPCR analysis to determine the expression of the mitochondriogenesis-related *NRF1, PGC-1A, TFAM,* and *CYBA* mRNAs*.* Data are mean ± SD (*n* = 6). **(G)** Glycolysis inhibition test with 2-deoxyglucose and a glucose deprivation test with glucose to measure the anti-metabolic stress ability of cells. Data are mean ± SD (*n* = 5).

Previously, miR-1291 was reported to affect tumor cell status. Here we first determined how miR-1291 specifically regulates tumor cell proliferation capacity and the metabolic state. The efficiency of transfection was measured by qPCR (Figure S2A). The aim of the WST-8 assay was to determine the viability of tumor cells, and BrdU incorporation was measured to directly investigate the role of miR-1291 in cell proliferation. Both the viability and proliferation of PANC-1 and MDA-MB-231 cells were markedly inhibited in the miR-1291-treated group (Figure 1A). Colony formation assays confirmed that overexpression of miR-1291 inhibited tumor cell colony formation (Figure 1B). The cell cycle and expression of cell cycle-related proteins were measured to investigate whether the reduction of cell proliferation by miR-1291 was related to the mechanistic alterations of the cell cycle and cell cycle-related proteins. miR-1291-transfected PANC-1 and MDA-MB-231 cells arrested the cell cycle in the G2/M phases and dramatically suppressed the expression of the cell cycle inducers CCNA, CCND and CCNE compared with that of the empty vector-expressing group (Figure 1C; Figure S4A).

Further studies were carried out to determine the role of miR-1291 in cancer cell metabolism. Under nutrient deficiency by culture medium deprivation, ATP production was attenuated after transfection with the miR-1291 expression plasmid (Figure 1D). Conversely, ROS levels were enhanced in miR-1291-transfected cells (Figure 1E). More importantly, mitochondrial biogenesis may be impaired by cell metabolic disorders. In miR-1291-transfected cells, the mRNA levels of *PGC-1A* and its downstream target genes *NRF1* and *TFAM*, and *CYTB* were measured. These mRNA levels were all reduced (Figure 1F). Cell survival under metabolic stress was further examined. Compared cells in the vector group, the sensitivity of cells with high expression of miR-1291 to glycolysis inhibition induced by 2-DG was significantly increased. The ability of miR-1291 expressing cells to resist proliferation inhibition induced by glucose deprivation was reduced (Figure 1G). Taken together, these data suggest that miR-1291 expression could inhibit cell proliferation and disrupt cell metabolism.

### miR-1291 reduces tumor cell tumorigenicity

**Figure 2:**
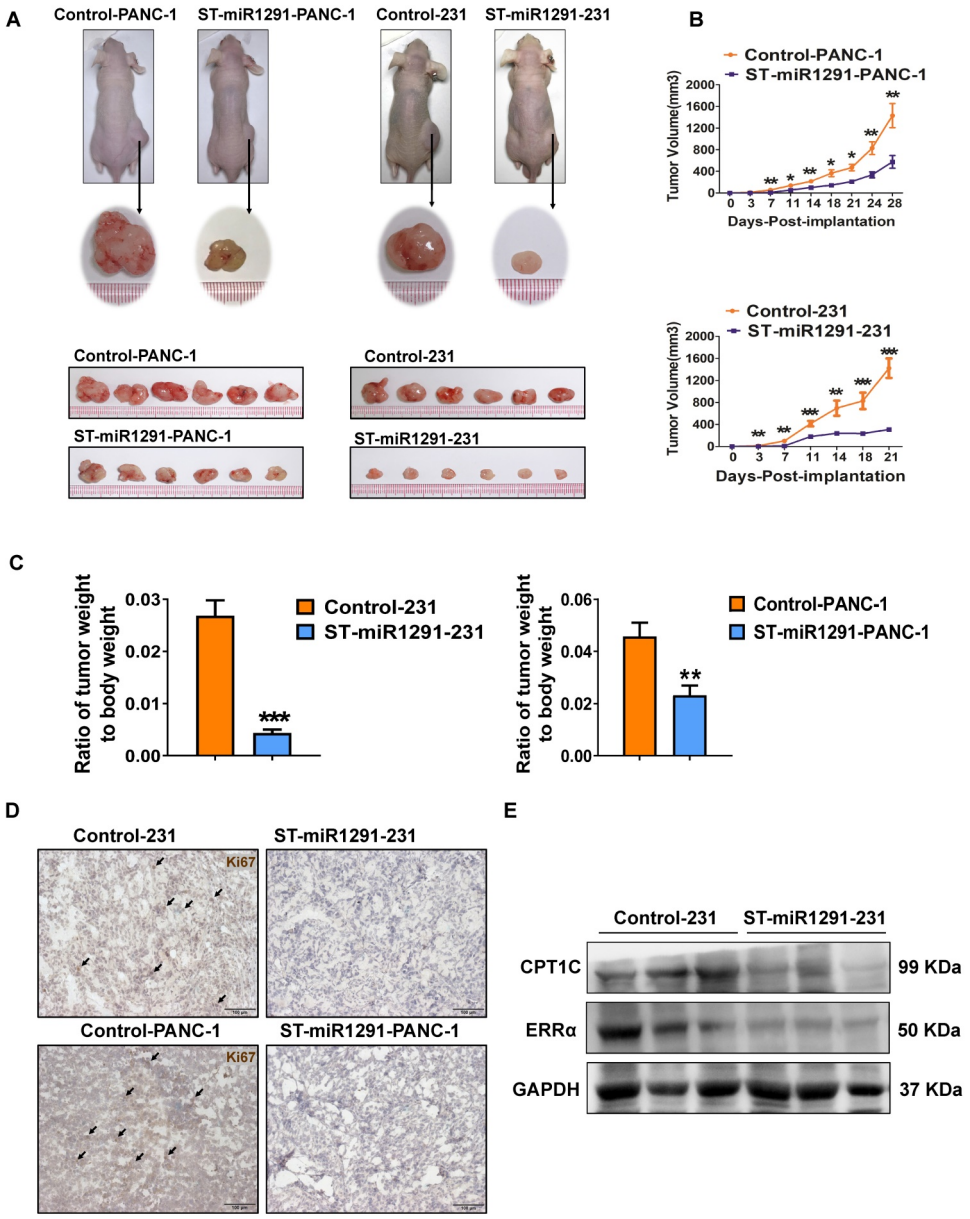
** miR-1291 reduces the tumor cell tumorigenicity. (A)** The images of xenograft tumor-bearing mice and tumors derived from PANC-1 and MDA-MB-231 cells infected with retroviruses expressing either miR-1291 or control vector (*n* = 8). **(B)** Subcutaneous xenograft tumors arising from PANC-1 and MDA-MB-231 cells were monitored for 4 weeks and 3 weeks, respectively. Tumor weights of mice in both groups. Tumor sizes are presented as mean ± SD over time (*n* = 8). **(C)** Comparison of the ratio of dissected tumor weights over body weights of mice. Data are mean ± SD (*n* = 8). **(D)** IHC analysis was used to determine Ki67 levels in tumor samples. **(E)** Western blot assay were used to measure ERRα and CPT1C protein in tumor samples.

Moreover, to define the impact of miR-1291 levels on the tumorigenesis, a xenograft tumor-bearing nude mouse model was established to evaluate the antitumor impact of miR-1291. ST-miR1291-PANC-1 or ST-miR1291-231 cells were injected subcutaneously into the right side of the nude mouse dorsum. Tumors derived from the ST-miR1291 cells were much smaller compared with the tumors in the control groups (Figure 2A). In addition, the tumors grew much more slowly in the ST-miR1291 cells than those in the parallel controls (Figure 2B), which were induced by miR-1291 activation. Significant differences in the ratios of tumor weight to body weight were observed (Figure 2C), indicating that miR-1291 suppresses tumor growth in xenograft mouse models. Moreover, a decreasing trend of Ki67 expression was observed in tumor samples of ST-miR1291-cells treated mice as revealed by IHC analysis, suggesting inhibition of cell proliferation by miR-1291 in tumors (Figure 2D). Western-blot analysis further indicated that the protein levels of ERRα and CPT1C were decreased in tumor samples from ST-miR1291-231 injected mice, consistent with the regulatory effects as noted below (Figure 2E).

### miR-1291 indirectly regulates CPT1C

**Figure 3:**
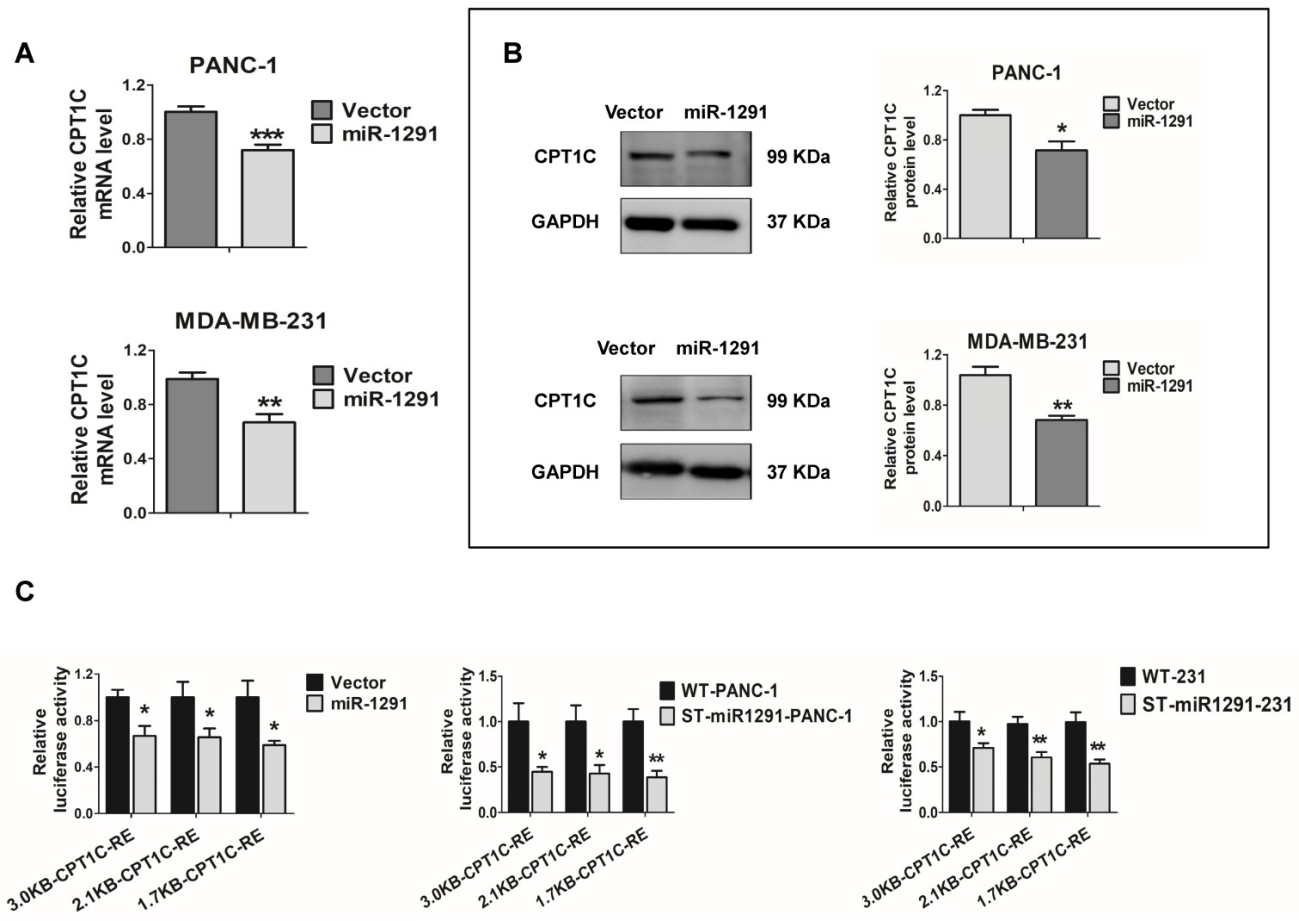
** miR-1291 indirectly regulates CPT1C. (A)** Expression of *CPT1C* mRNA in PANC-1 and MDA-MB-231 cells after transfection with miR-1291. Data are mean ± SD (*n* = 6). **(B)** Western blot assay was used to detect expression of CPT1C after transfection with miR-1291. Band intensity was evaluated by Quantity one software. Data are mean ± SD (*n* = 3). **(C)** Luciferase reporter gene assays were conducted in HEK-293T cells treated with different *CPT1C* reporter plasmids to define the impact of miR-1291. ST-miR1291-PANC-1 and ST-miR1291-231 cell lines were also used to verify the effect of miR-1291 on luciferase activities of the *CPTIC* promoters. Data are mean ± SD (*n* = 5).

The *CPT1C* mRNA and CPT1C protein levels were measured in the miR-1291 over-expressing cells. *CPT1C* mRNA was decreased after transfecting the miR-1291 plasmid into the two cell lines (Figure 3A). Western-blot analyses also showed an obvious decrease in CPT1C protein levels as a result of miR-1291 expression (Figure 3B). The effect of miR-1291 on the *CPT1C* promoter was then determined. Over-expression of miR-1291 significantly down-regulated the activity of *CPT1C* promoter-luciferase constructs of different lengths (Figure 3C). Stably transfected cell lines (ST-miR1291-PANC-1 and ST-miR1291-231) were also applied for the *CPT1C* promoter-luciferase reporter gene assays, and similar tendencies were observed (Figure 3C). The miR-1291 levels of these ST-miR1291 cell lines were also measured (Figure S2A). However, the prediction analysis and luciferase reporter assays of *CPT1C* mRNA-3'UTR showed negative results (Figure S3A) indicating that the effect of miR-1291 on CPT1C was indirect. Collectively, these data indicate that CPT1C levels are inversely correlated with miR-1291 levels, suggesting that there must be a crucial regulatory factor between miR-1291 and CPT1C.

### miR-1291 targets the ERRα pathway

**Figure 4:**
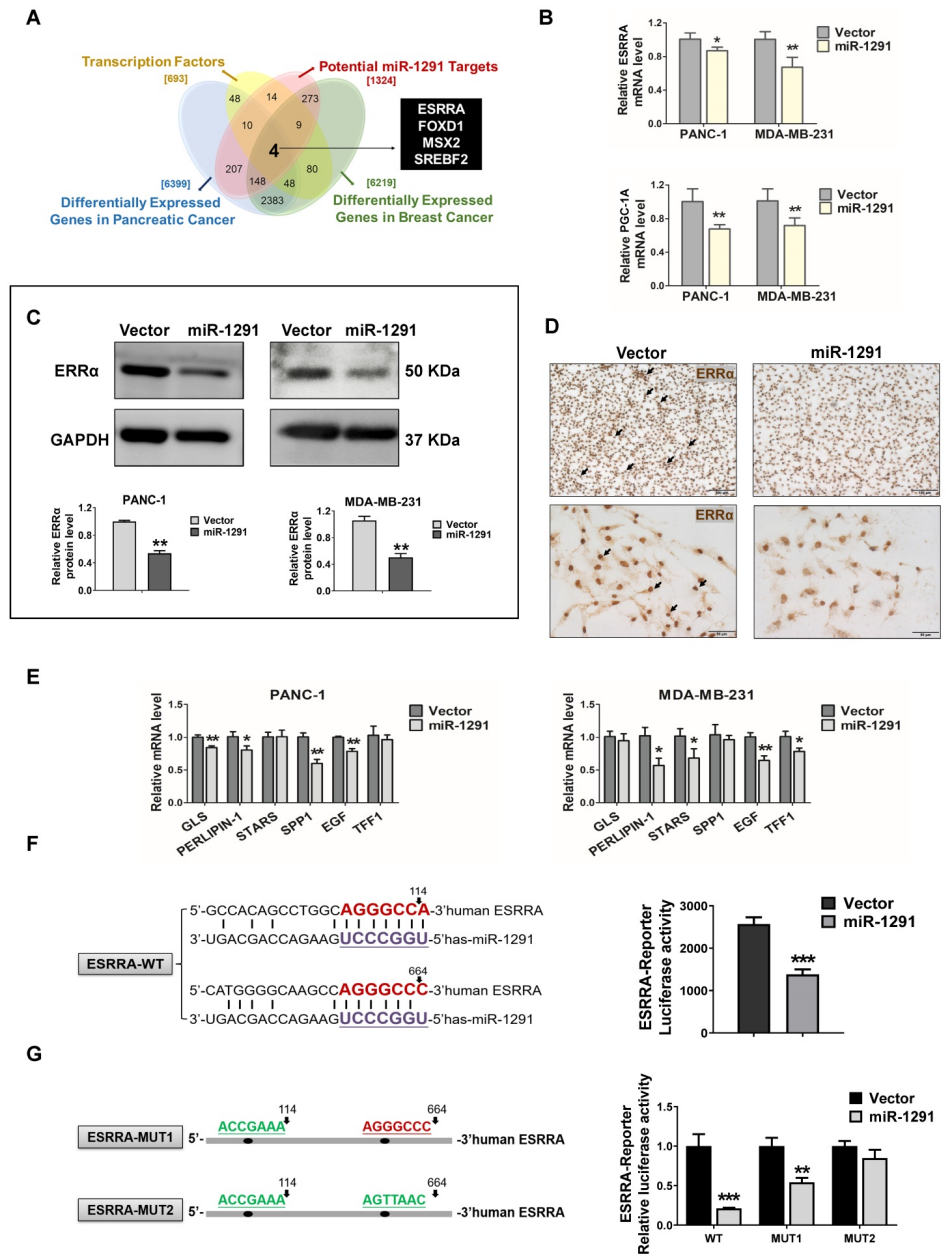
** miR-1291 targets the ERRα pathway. (A)** The Venn diagram displayed the overlaps between four different gene sets.** (B)** Levels of *ESRRA* and *PGC-1A* mRNAs in PANC-1 and MDA-MB-231 cells after transfection with miR-1291. The data are the mean ± SD (*n* = 6). **(C)** Western blot analysis of ERRα after transfection with miR-1291. Data are mean ± SD (*n* = 3). **(D)** MDA-MB-231 cell lines were stained for ERRα.** (E)** ERRα target gene mRNA levels measured under the same conditions described above. Data are mean ± SD (*n* = 6). **(F)** Bioinformatics analysis revealing two putative MRE sites for miR-1291 within the 3'UTR of *ESRRA* transcript. The seed sequence of miR-1291 is underlined. *ESRRA* 3'UTR luciferase reporter activities were determined. Data are mean ± SD (*n* = 5). **(G)** The sequence of *ESRRA* 3'UTR bearing the mutated MRE sites is shown. The *ESRRA* 3'UTR luciferase reporter activities were detected with two *ERRα*-Reporter-MUT plasmids. Data are mean ± SD (*n* = 5).

Based on meta-analysis of multiple types of studies by using the GEO database, 6219 and 6399 significantly highly expressed genes were explored in breast and pancreatic cancer tissue samples compared with normal tissues, respectively (Table S5). Through intersection with these differentially expressed genes, the potential miR-1291 target database and transcription factor database was generated to further refine the results and four gene candidates, *ESRRA, FOXD1, MSX2* and *SREBF2*, were uncovered (Figure 4A)*.* By analyzing the matching degree of their specific DNA-binding site sequences to *CPT1C* promoter sequences, *ESRRA* showed a significantly higher degree of binding ability than the other three.

Thus, efforts were directed to clarify how miR-1291 might regulate ERRα expression in cancer cells. Both the *ESRRA* mRNA and its coactivator *PGC-1A* mRNA levels were significantly down-regulated in PANC-1 and MDA-MB-231 cells after transfecting the miR-1291 expression plasmid (Figure 4B). Concurrently, the lower levels of ERRα and PGC-1α protein expression were determined in miR-1291-expressing cells (Figure 4C, Figure S4D). IHC images confirmed that the positive staining of ERRα was decreased in the miR-1291-overexpressing MDA-MB-231 cells (Figure 4D). A series of mRNAs encoded by ERRα target genes were measured, and most expression was reduced after forced expression of miR-1291 (Figure 4E). At the same time, the effects of miR-1291 on ERRα-CPT1C pathway were also confirmed using the miRNA inhibition strategy and the efficiency of miR-1291 inhibitor was described (Figure S2A). Downward trends of ERRα protein levels and CPT1C mRNA expression were observed with miR-1291 inhibition treatment (Figure S3C). A luciferase reporter assay showed that increased miR-1291 expression was able to significantly reduce *ESRRA* 3'UTR activity (Figure 4F). To evaluate the accuracy of the two miR-1291 binding elements, another reporter assay illustrated that the induction of miR-1291 triggered a decrease of luciferase activity from the WT-*ERRα* reporter plasmid but produced a smaller or no change in the luciferase activity from the MUT-*ERR*α reporter plasmid (Figure 4G). These results indicate that miR-1291 regulates ERRα expression negatively by direct binding to the 3'UTR seed regions in the *ESRRA* mRNA.

### *CPT1C* is an ERRα target gene

**Figure 5:**
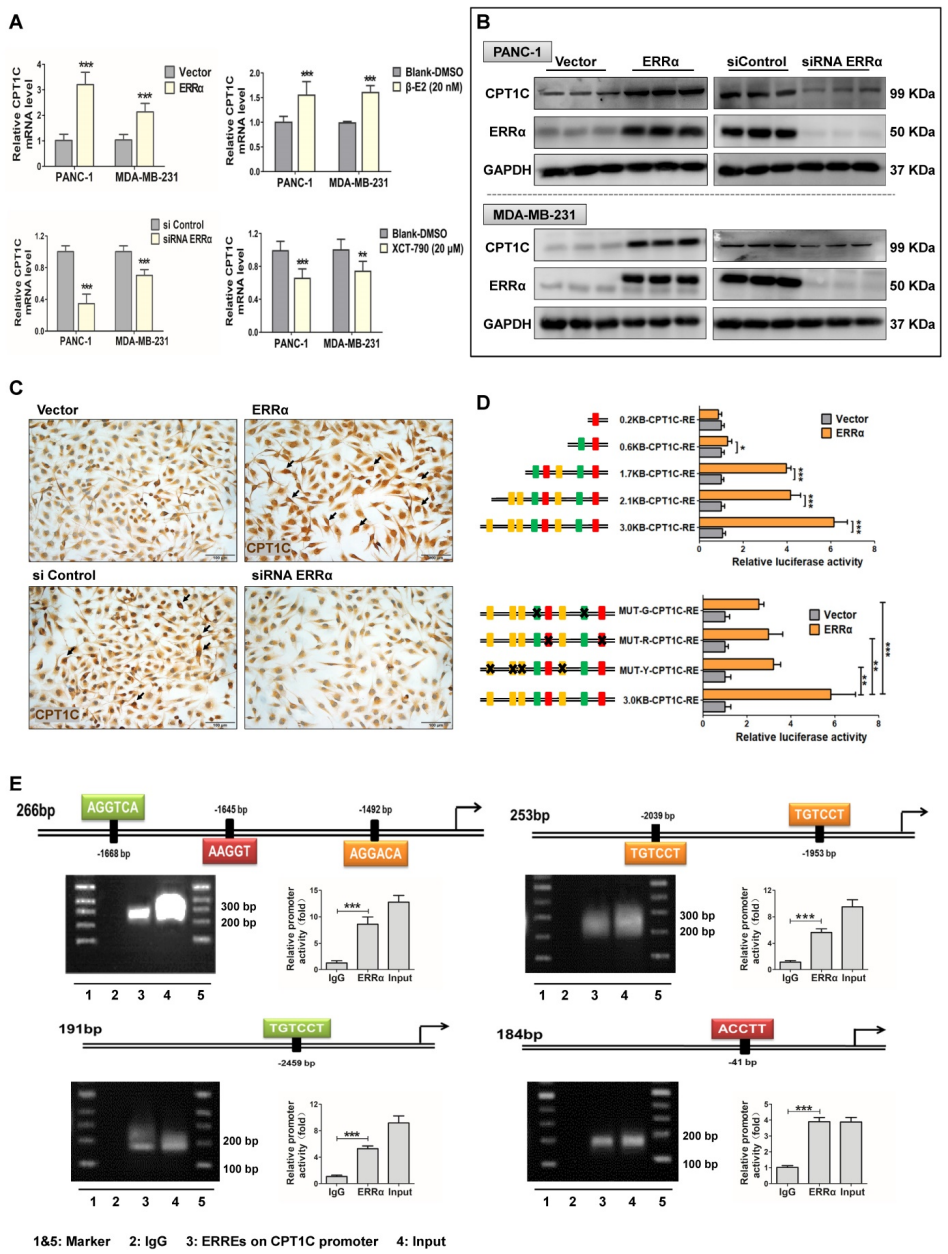
** ERRα regulates CPT1C expression and activates CPT1C transcription directly. (A)** Expression of *CPT1C* mRNA in PANC-1 and MDAMB-231 cells after modulating ERRα expression and activity with the pENTER-ERRα plasmid and an agonist β-E2 (20 nM), as well as ERRα siRNA and an inhibitor XCT790 (20 μM). Data are mean ± SD (*n* = 6). **(B)** Western blot analysis ERRα and CPT1C after perturbation of ERRα. Data are mean ± SD (*n* = 3). **(C)** MDA-MB-231 cell lines stained for CPT1C. **(D)** Luciferase reporter gene assays were conducted in HEK-293T cells to compare transcriptional activities among plasmids within different lengths of *CPT1C* promoter regions or mutated ERRE sequences. MUT-G-CPT1C: ERRE-2-GREEN were mutated. MUT-R-CPT1C: ERRE-1-RED were mutated. MUT-Y-CPT1C: ERRE-3-YELLOW were mutated. Data are mean ± SD (*n* = 5). **(E)** MDA-MB-231 cells were treated with the pENTER-ERRα plasmid for 48 h, and ChIP analysis was performed. The DNA samples from the precipitation were amplified with PCR.

ERRα is an essential transcription factor controlling genes involved in energy metabolism and mitochondrial function which is in line with the function of CPT1C. Sequence analysis reveals that the *CPT1C* promoter has consensus ERRα binding sites, thus indicating that ERRα might regulate the *CPT1C*. *CPT1C* mRNA and CPT1C protein levels were measured in PANC-1 and MDA-MB-231 cells after forced expression of ERRα with the pENTER-ERRα plasmid and activation with the agonist β-E2, and after down-regulating ERRα with siRNA and treatment with the ERRα inhibitor XCT-790. The efficiency of these plasmids, siRNAs and drugs to control ERRα levels are described in Figure S2B. Appropriate doses of drugs were chosen according to cytotoxicity testing using the WST-8 assay (Figure S2C). *CPT1C* mRNA was changed in an ERRα-dependent manner (Figure 5A) with similar results observed at the protein level (Figure 5B, Figure S4B). CPT1C protein status in MDA-MB-231 cells was also examined using IHC analysis and a correlation between ERRα and CPT1C expression was found (Figure 5C). These data strongly demonstrate that ERRα controls CPT1C expression.

To investigate whether ERRα directly regulates CPT1C, luciferase reporter gene and ChIP assays were performed. First, 8 potential ERRE binding elements were predicted in the *CPT1C* 3.0 kb promoter region by bioinformatics analysis and divided into three elements (ERRE1-RED, ERRE2-GREEN and ERRE3-YELLOW) based on the diversity of the sequences (Figure S3D). Several *CPT1C* promoter regions with different lengths or mutated sites were cloned into the pGL3-basic vector. The luciferase activity assay suggested that a 3.0 kb region upstream of CPT1C core coding domain and ERRα were necessary for ERRα regulation of the *CPT1C* promoter. In addition, the transcriptional activity of the 2.1 kb and 1.7 kb *CPT1C* promoters, which still contained seven or five ERREs, could be activated by the transfection of ERRα. Serial deletion analysis showed that the transactivation in 0.6 kb and 0.2 kb length CPT1C promoter constructs was markedly decreased or abolished (Figure 5D). Moreover, three plasmids with mutated ERREs sequences exhibited significantly low luciferase activity compared with the normal 3.0 kb promoter plasmid (Figure 5D). ERRα's binding to the *CPT1C* promoter was further verified using ChIP assays in which the efficacy of micrococcal nuclease to cutting the DNA fragments was assessed (Figure S3E). In vitro binding experiments revealed efficient recruitment of ERRα to the putative ERREs (Figure 5E). These results indicate that ERRα regulates CPT1C directly by binding to several different sites on the *CPT1C* promoter. From the above data, the existence of the miR-1291-ERRα-CPT1C axis was verified. Thus, ERRα could be the regulatory link between miR-1291 and CPT1C.

### ERRα influences cancer cell proliferation and metabolism

**Figure 6:**
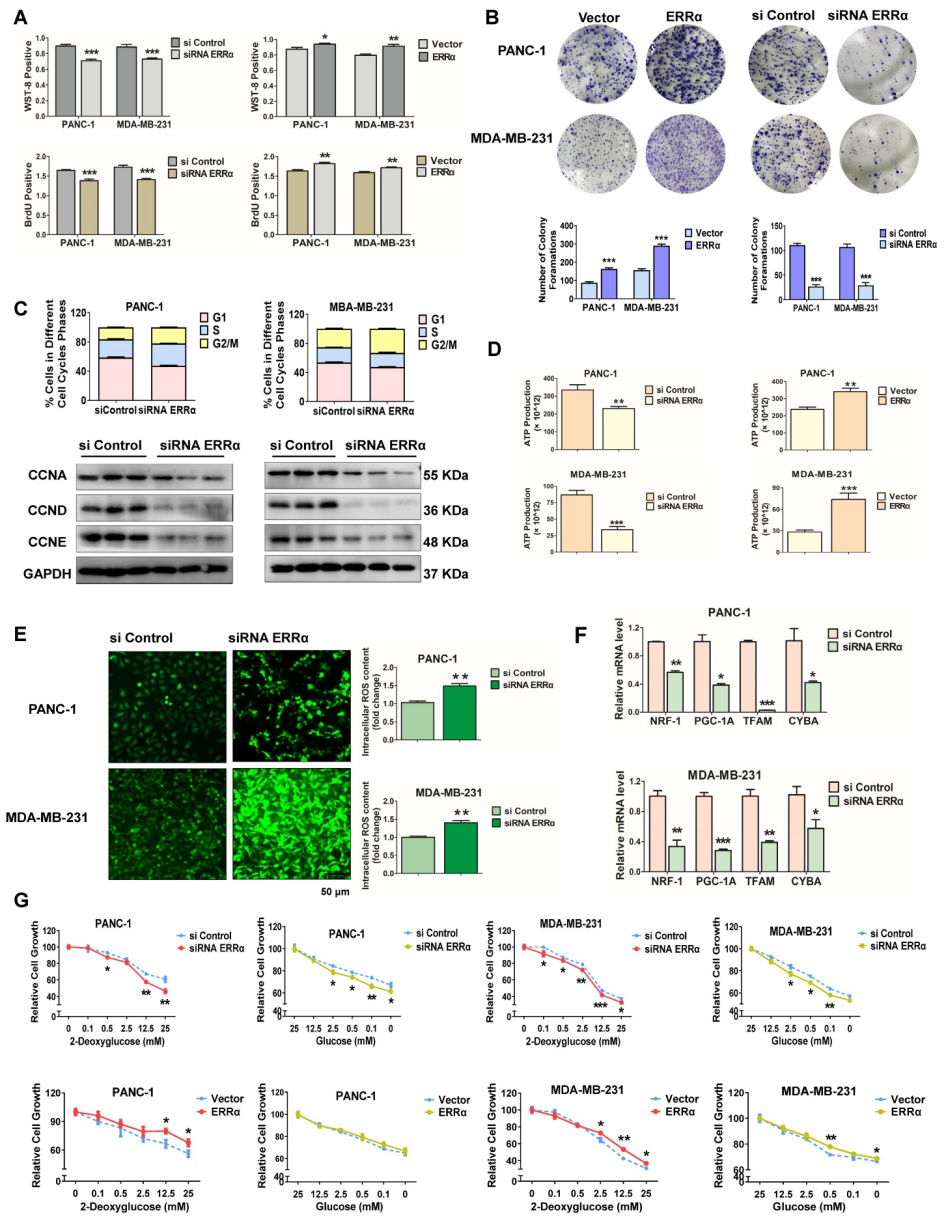
** ERRα modulates cancer cell proliferation and metabolism. (A)** WST-8 and BrdU assays were performed to examine the viability and proliferation capacity of PANC-1 and MDA-MB-231 cells after modulation of ESRRA levels by siRNA and expression plasmid. The data are the mean ± SD (*n* = 5). **(B)** After transfection with the ERRα expression plasmid and siRNA ERRα, cells were cultured for an additional two weeks and stained with Diff-Quik to determine colony formation capacity. **(C)** The cell cycle was determined by flow cytometry after the transfection of ERRα siRNA. The protein levels of cell cycle-related proteins, such as cyclin A/D/E were determined by immunoblot analysis after transfection with ERRα siRNA. Data are mean ± SD (*n* = 3). **(D)** ATP production in both high ERRα and low ERRα cells was detected. Data are mean ± SD (*n* = 5). **(E)** The intracellular accumulation of ROS in two cell lines was examined following the depletion of ERRα. Data are mean ± SD (*n* = 5). **(F)** Expression of mitochondriogenesis-related mRNAs *NRF1, PGC-1A, TFAM*, and *CYBA.* Data are mean ± SD (*n* = 6). **(G)** A glycolysis inhibition test with 2-deoxyglucose and a glucose deprivation test with glucose were performed to measure the anti-metabolic stress ability of tumor cells. Data are mean ± SD (*n* = 5).

Since the data mentioned above indicate a close relationship between miR-1291 and ERRα, the potential that ERRα modulates cell proliferation and metabolic conditions was examined. ERRα-deficient PANC-1 and MDA-MB-231 cells had reduced levels of survival and proliferation, while the viability and proliferation ability increased after forced expression of ERRα in both cell lines (Figure 6A). Colony formation assays revealed that ERRα promoted cellular colony formation ability, and ERRα inhibition significantly reduced the form of macroscopic colonies (Figure 6B). After transfection with siRNA to* ESRRA* mRNA, tumor cells demonstrated higher numbers of cells in the G2/M phase vs those in the siControl group. The expression of cell cycle-related proteins, such as CCNA, CCND and CCNE were also suppressed (Figure 6C, Figure S4C), consistent with the results of miR-1291. On the other hand, ATP synthesis decreased in cells deficient in ERRα. ERRα expression increased ATP generation and siRNA knockdown of *ESRRA* mRNA decreased ATP generation (Figure 6D). Correspondingly, ERRα knockdown elevated the ROS levels as evidenced by a cellular reactive oxygen metabolism assay (Figure 6E). Furthermore, after the depletion of ERRα, the *PGC-1A* and its downstream *NRF1, TFAM* mRNAs as well as mtDNA subunit *CYTB* mRNA were reduced (Figure 6F). Finally, the capacity of cell survival under metabolic stress was determined. Cancer cells with ERRα knockdown showed a higher sensitivity to 2-DG or glucose deprivation compared to the sensitivity of the control cells (Figure 6G). After overexpressing ERRα, the cells' tolerance to proliferation inhibition was examined. Proliferation inhibition induced by either 2-DG or glucose deprivation was increased (Figure 6G). Hence, these data illustrate that the depletion of ERRα decreases the capacity to resist metabolic stress of PANC-1 and MDA-MB-231 cancer cells, and results in the inhibition of proliferation and metabolism; while forced ERRα overexpression improves metabolic status and promotes cell proliferation.

### miR-1291-ERRα-CPT1C axis has synergistic regulation on tumor cell proliferation and metabolism

**Figure 7:**
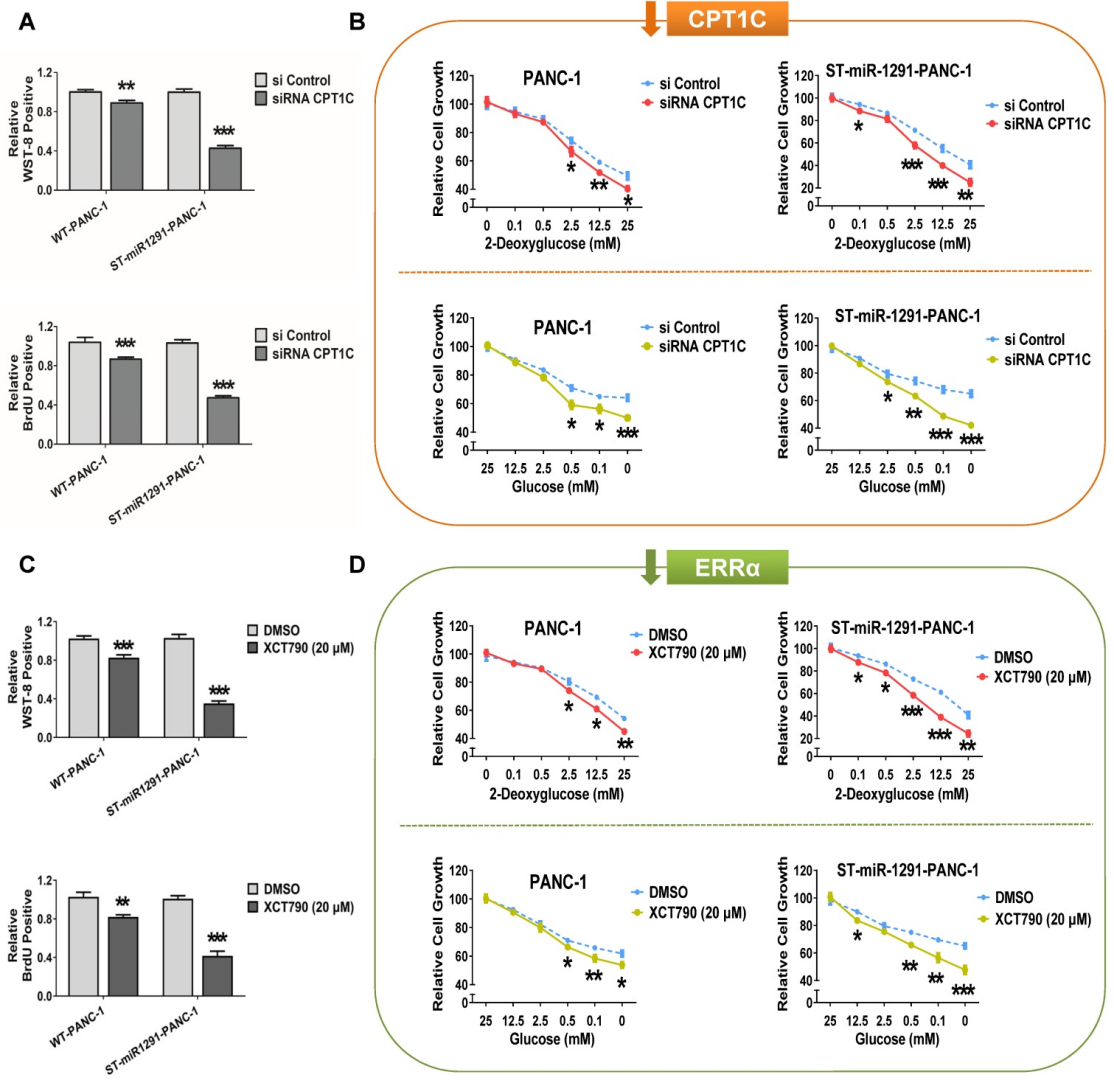
** Synergistic regulation of miR-1291-ERRα-CPT1C signaling on tumor. (A)** WST-8 and BrdU assays were performed to examine the effect of low CPT1C expression on the viability and proliferation capacity of WT and ST-miR1291 tumor cells. Data are mean ± SD (*n* = 5). **(B)** Glycolysis inhibition tests with 2-deoxyglucose and glucose deprivation tests with glucose were performed to measure the depletion of CPT1C expression on the anti-metabolic stress ability of WT and ST-miR1291 tumor cells. Data are mean ± SD (*n* = 5). **(C)** WST-8 and BrdU assays were performed to examine the influence of ERRα inhibition on the viability and proliferation capacity of WT and ST-miR1291 tumor cells. Data are mean ± SD (*n* = 5). **(D)** Glycolysis inhibition tests with 2-deoxyglucose and glucose deprivation tests with glucose were performed to measure the impact of reduction of ERRα expression on the anti-metabolic stress ability of WT and ST-miR1291 tumor cells. Data are mean ± SD (*n* = 5).

A previous study has revealed a vital role of CPT1C in cancer cell proliferation, metabolism and senescence-associated mitochondrial dysfunction 11. Based on the above findings, synergistic regulation of the miR-1291-ERRα-CPT1C axis on tumor progression was examined. In cases of miR-1291 background levels, the sensitivity of cells to the alteration of cell proliferation and metabolism caused by different expression levels of CPT1C or ERRα was compared. The efficiency of plasmids and siRNA to control CPT1C expression are described (Figure S2D). Overexpression of CPT1C in ST-miR1291 cells significantly increased cell proliferation compared with the levels in WT cells (Figure S5A&S6A). In contrast, CPT1C silencing suppressed cell survival more in the ST-miR1291 cell lines (Figure 7A, Figure S7A). Compared to that of WT cells, ST-miR1291 cells were more sensitive to the increase in the cell survival rate caused by increased CPT1C expression under metabolic pressure (Figure S5B&S6B). Depletion of CPT1C increased the vulnerability of ST-miR1291cells to metabolic inhibition (Figure 7B, Figure S7B). Similar tendencies of the effect of ERRα on the axis were found. Low ERRα levels exhibited better antitumor sensitivities in ST-miR1291 cells (Figure 7C&D, Figure S7C&D). Further activation of ERRα enhanced the survival and metabolism in ST-miR1291 cells compared to WT cells (Figure S5C&D, Figure S6C&D), even if these trends were not very significant. These data suggest a synergistic regulation of the miR-1291-ERRα-CPT1C axis on the proliferation and metabolism of cancer cells.

## Discussion

Malignancies are still the most lethal diseases despite several decades of therapeutic research and thus it is imperative that novel treatments for cancer continue to be developed. miRNAs are a type of noncoding RNAs involved in critical biological and physiological processes. In recent years, their deregulation and functional implications have been well documented in a large number of human diseases, especially cancer 24,25. Supported by the evidence of miRNAs' critical role in cancer, they could have therapeutic potential. Overexpression or downregulation of miRNAs changes the internal cellular pathways that contribute to tumor cellular metabolism status, carcinogenesis tumor progression and metastasis. For example,* in vitro* and* in vivo* studies have shown that miR-34a, Let-7, miR-21, miR-23 and miR-122 may be promising targets for miRNA-based therapy for human cancers 26-30.

miR-1291 has received increasing attention in the study of cancer. In previous studies, miR-1291 levels were significantly lower in pancreatic tumor tissues, esophageal squamous cell carcinoma and renal cell carcinoma specimens compared with their levels in control tissues or cell lines 4,31,32. Prior studies also revealed that the restoration of miR-1291 expression inhibits cancer cell proliferation, invasion, tumorigenesis and the cell metabolome 2,4. Recombinant fully-humanized bioengineered tRNA/miR-1291 was produced in large quantities, and the therapeutic potential of this miR-1291 prodrug as a monotherapy for pancreatic cancer has gained increasing attention 1,33. In addition, certain targets have been identified for miR-1291, including the efflux transporter MRP1, the glucose transporter GLUT1, the essential signaling pathway for cell differentiation and fatty acid oxidation, FOXA2-AGR2, the mucus maker MUC1, the endoplasmic reticulum stress sensor IRE1α and GPC3 3-5,32,34,35, all of which are critical for cell energy metabolism. The above information may indicate that the mechanism of action of miR-1291 as a potential antitumor factor is likely to be achieved by comprehensively inhibiting cellular metabolic pathways. Therefore, the present study illuminated a new downstream regulatory mechanism for miR-1291 at the metabolic level.

As a member of the CPT family, CPT1C has emerged as a potential therapeutic target in various types of cancer, such as breast cancer and neuroblastoma 8,10,36. Compared to normal tissue, clinical tissue samples from patients with multiple cancers, especially pancreatic cancer and breast cancer, showed high CPT1C expression (37,38, and unpublished data). These data provided clinically relevant support for the present study. CPTs participate in the first vital step of fatty acid metabolism, the reversible transesterification of acyl-CoA esters and carnitines to form acyl-carnitine esters in the mitochondrial membrane 39. Cancer cells can tolerate energy stress by increasing fatty acid synthesis 40,41. The majority of tumors maintain survival and viability by consuming fatty acids, and the FAO process is the primary energy source 42. This is due to the fact that CPT1C, which is closely related to lipid metabolism, is induced under the conditions of glucose deprivation or hypoxia 10, 36. Moreover, CPT1C may be a key element of mitochondrial dysfunction-associated tumor cellular proliferation and senescence and functionally differs from the other three subtypes CPT1A, CPT1B and CPT2 11,12. Referring to the upstream regulatory mechanisms of CPT1C, the AMPK-ACC-CPT1 signaling pathway is recognized as a key regulator of FAO, and the expression of CPT1C can be induced by AMPK activation in a p53-dependent manner 10,43. Moreover, CPT1C is an important PPARα target that may be involved in cellular proliferation and senescence 22. However, the upstream regulatory mechanisms that lead to a series of CPT1C-dependent cell events remain unclarified, and no studies have reported connections between miRNAs and CPT1C. The current work revealed the molecular mechanism by which miR-1291 and CPT1C synergistically regulate the proliferation and metabolism of tumor cells.

Although the activation of miR-1291 decreased CPT1C expression at both the mRNA and protein levels, both a prediction analysis and a reporter assay of *CPT1C* 3'UTR showed negative results, which indicated that miR-1291 did not directly target CPT1C. Thus the next step was to seek the possible link between miR-1291 and CPT1C. Both in the current and previous studies, the breast cancer cell line MDA-MB-231 and the pancreatic cancer cell line PANC-1 were used for the specific expression of CPT1C and miR-1291 in pancreatic and breast cancer tissues, as mentioned above. Thus, the screening of genes that are highly expressed in both pancreatic and breast tumor tissues supported by GEO database was performed. GEO is a public functional genomics data repository, which contains vast array and sequence based data. The meta-analysis process is able to integrate all results from previous studies, which made our targets precise and reliable. On the other hand, we expected the target gene to satisfy two conditions: its mRNA-3'UTR region should have a good potential binding capacity with miR-1291, and belong to a transcription factor family that directly regulates *CPT1C* transcription. The Targetscan database and the JASPAR database helped us acquire “Prediction of miRNA Targets” or “Collection of transcription factor DNA-binding preferences” information. Among thousands of candidate targets, *ESRRA, FOXD1, MSX2* and *SREBF2* were investigated. In addition, the specific DNA binding site sequence of *ESRRA* was found to match the *CPT1C* promoter sequence better than the other three genes. As expected, ERRα, a key factor regulating fatty acid metabolism, is the bridge linking miR-1291 and CPT1C. ERRα is not merely a modulator of energy homoeostasis but rather has distinct activity that could contribute to the pathogenesis and development of cancers 13,16. The current study also revealed other potential pathways such as *CPT1C* as a downstream target gene of ERRα via the AMPK/PGC-1β pathway and 39,44. Thus, the miR-1291-ERRα-CPT1C axis was proposed.

The current work confirmed that miR-1291 inhibited the proliferation capacity, metabolic status and tumorigenicity of the pancreatic cancer cell line PANC-1 and the breast cancer cell line MDA-MB-231. WST-8 and BrdU assays were performed to measure cell survival indirectly and proliferation directly. Inhibition of cell proliferation mainly manifests as a decreased colony formation ability and cell cycle arrest. The data obtained from these two assays are in agreement with the conclusion that miR-1291 modulates proliferation and agree with previous studies 4. Growing evidence has shown that cancer cells tend to acquire more vigorous metabolic rates due to reprograming of metabolic pathways involved in glutamine, glucose synthesis and those involving lipogenic or lipolytic enzymes such as FAO-related mitochondrial metabolism to generate more the metabolites needed for their high biosynthetic and bioenergetic demands 45,46. The low ATP production and concomitant ROS accumulation observed in the present study indicated a collapse of mitochondrial function and the metabolic capacity of tumor cells. On the other hand, the decreasing biosynthesis ability of mtDNA (mitochondrial DNA), which has relatively high exposure, reflects the impairment of the normal metabolism to some extent 47,48. Consistently, the *PGC-1A* mRNA levels and downstream signaling molecule mRNAs *NRF1* and *TFAM* were decreased, and the *CYTB* mRNA levels were also subsequently lowered, which affected mtDNA replication. In addition, malignant tumor cells often show a strong tolerance to metabolic stress. Thus, the assessment of the viability of cells exposed to 2-DG or under glucose-starved conditions was determined to judge their resistance to metabolic stress. Furthermore, the tumorigenicity analysis of the miR-1291-based xenograft tumor model was repeated, and the MDA-MB-231 cell line was added. This minimized interindividual variability and some other confounding factors. Consistently, the results obtained from the present and previous studies indicate that miR-1291 acts as a tumor suppressor, and MDA-MB-231 cells reveals a higher sensitivity to the tumorigenic alterations induced by miR-1291 2,4.

We further measured the effect of ERRα knockdown on tumor cell proliferation and metabolism and the results were similar with those caused by high expression of miR-1291, but were not exactly the same. For example, miR-1291 inhibits GLUT-1 expression 5, which is a transporter directly related to glucose uptake. Thus, compared with ERRα silencing, overexpression of miR-1291 displayed greater sensitivity to glycolysis. On the other hand, ERRα, as a vital miR-1291 target protein, is required for the activation of mitochondrial genes as well as increased mitochondrial biogenesis 49. Thus, it is reasonable that mitochondrial biosynthesis and mitochondrial function-related genes were significantly reduced after transfection with siRNA ERRα. As miR-1291 is upstream of ERRα, the biological effect on mitochondria is relatively delayed after miR-1291 overexpression.

Further studies were done to establish the miR-1291-ERRα-CPT1C axis. Sequences information revealed two putative MRE sites for miR-1291 within the 3'UTR of *ESRRA* transcript (Figure S3B). Luciferase reporter gene assays of the *ERSSA* 3'UTR were conducted, and specific mutant luciferase assays aimed at putative MREs were made on the basis of bioinformatic predictions. miR-1291 decreased luciferase activity, and when mutations that disrupt the binding of *ERSSA* 3'UTR to the miR-1291 “seed” sequence were introduced into *ERRα*-MUT reporter plasmids, the luciferase activity was almost completely abolished. These results indicate that *ESRRA* mRNA is a bonafide target gene of miR-1291. In addition, ERRα was found to directly regulate the *CPT1C* gene. Eight typical ERRE binding elements were uncovered. A series of ERRE sites were found and luciferase analysis suggested that the *CPT1C* promoter fragments consisted of a number of binding sites having a strong binding capacity toward ERRα. Until the number of binding sites was drastically reduced to one or two, ERRα was no longer able to activate the reporter gene transcription. The results of the mutation luciferase reporter gene assays indicated that the site designated ERRE-2-GREEN may be most conserved and crucial to the binding effect. ChIP experiments were conducted proving the direct regulatory effect of ERRα on *CPT1C*.

As noted above, a variety of energy metabolism-related signaling factors, metabolic enzymes and transporters have been confirmed as downstream pathways of miR-1291 which has attracted increasing attention as an antitumor target or potential cancer treatment strategy. Therefore, it is necessary to explore how ERRα and CPT1C, two known key molecules that can respectively affect the proliferation and metabolism of tumor cells, play an important role in the action of miR-1291. In addition, it is necessary to explore how this altered action of miR-1291 affects the fate of tumor cells after the existence of the miR-1291-ERRα-CPT1C axis is confirmed. Therefore, ERRα or CPT1C expression changes would be expected to have a greater impact due to the low cell functions caused by the high expression of miR-1291. WST-8 or BrdU positives after transfection were normalized to “100%” because of the different growth rate of ST-miR-1291 cells and WT cells (Figure S5E). Then the proliferation inhibition effects of these two types of cells were compared at the same level, respectively. Tumor cells with high miR-1291 expression would be more sensitive to inhibition of proliferation and the decrease of the ability to resist metabolic pressure caused by the depletion of ERRα or CPT1C. On the other hand, rescue experiment showed the opposite result (Figure S5A-D, S6). These data further reflect the significance of the ERRα-CPT1C relationship, which is downstream of miR-1291, in the antineoplastic characteristic of miR-1291. Thus, the miR-1291-ERRα-CPT1C axis could coordinate with the synergistic regulation of tumor cell proliferation and metabolism, and the combination of miR-1291 with other drugs or treatment methods related to ERRα and CPT1C may be possible.

Taken together, this study described that high miR-1291 expression inhibits cell proliferation and cell metabolism, and reduces the tumorigenicity of cancer cells. Moreover, this study demonstrated that a unique negative regulation exists between miR-1291 and ERRα, and that *CPT1C* is a novel ERRα target gene. The miR-1291-ERRα-CPT1C axis concept was proposed to clarify the mechanism of miR-1291 as a potential antitumor agent. The effects of ERRα expression on tumor cell proliferation and metabolism were also explored to make the system more comprehensive and persuasive. Finally, the vital function and efficiency of ERRα and CPT1C in the miR-1291 integrated axis were illustrated. Collectively, these results support a crucial role of the ERRα-CPT1C pathway in the anti-tumor effect of miR-1291, and suggest a new anticancer strategy involving the miR-1291-ERRα-CPT1C axis.

## Availability of Data and Materials

The stem-loop sequence and other information of human micro-RNA-1291 are available in miRbase website. The Gene expression data are available in Gene Expression Omnibus (GEO) database. The coding sequences of the ERRα (ESRRA) mRNA-3'UTR segment consisting of miR-1291 MRE (miRNA response elements) sites are available in the Targetscan database. The authors declare that all the data supporting the findings in this study are available from the corresponding author through reasonable request.

## Abbreviations

2-DG: 2-Deoxyglucose
3'UTR: 3'-untranslated region
ChIP: chromatin immunoprecipitation
CPT1C: carnitine palmitoyltransferase 1C
ERRα: estrogen-related receptor α
ERREs: ERR responsive elements
FC: fold change
FOXA2: fork head box protein A2
GEO: Gene Expression Omnibus
GLUT1: glucose transporter 1
IHC: immunohistochemistry
MRE: miRNA response elements
MRP1: multidrug resistance-associated protein 1
mtDNA: mitochondrial DNA
SNORA34: small nucleolar RNA H/ACA box 34
ST: stably transfected
