# Supplementary material for: A novel miR-1291-ERRα-CPT1C axis modulates tumor cell proliferation, metabolism and tumorigenesis
Source: Theranostics. 2020 Jun 1;10(16):7193–210. doi: 10.7150/thno.44877 (PMC7330864; doi:10.7150/thno.44877)
Supplement: Supplementary file 1 — Supplementary figures and table S1-3. [file thnov10p7193s1.pdf]

A novel miR-1291-ERRα-CPT1C axis modulates tumor cell proliferation, metabolism and tumorigenesis

Yixin Chen[1], Yanying Zhou[1], Fangwei Han[2], Yingyuan Zhao[1], Meijuan Tu[3], Yongtao Wang[1], Can Huang[1], Shicheng Fan[1], Panpan Chen[1], Xinpeng Yao[1], Lihuan Guan[1], Ai-Ming Yu[3], Frank J. Gonzalez[4], Min Huang[1], Huichang Bi[1,*]

Supplementary Figures

Figure S1: The workflow of the Meta-analysis.

Figure S2: The efficiency of plasmids, miRNA inhibitor, siRNAs and drugs.

Figure S3: The effect of miR-1291 on CPT1C-Reporter luciferase activity.
The regulation of miR-1291 on ERRα and CPT1C in miR-1291 inhibition strategy.
The information of binding sites and the efficacy of micrococcal nuclease.

Figure S4: The statistical results of protein expression.

Figure S5: The rescue experiment of miR-1291-ERRα-CPT1C axis synergistic regulation on PANC-1 cells.

Figure S6: The rescue experiment of miR-1291-ERRα-CPT1C axis synergistic regulation on MDA-MB-231 cells.

Figure S7: Synergistic regulation of miR-1291-ERRα-CPT1C signaling on tumor.

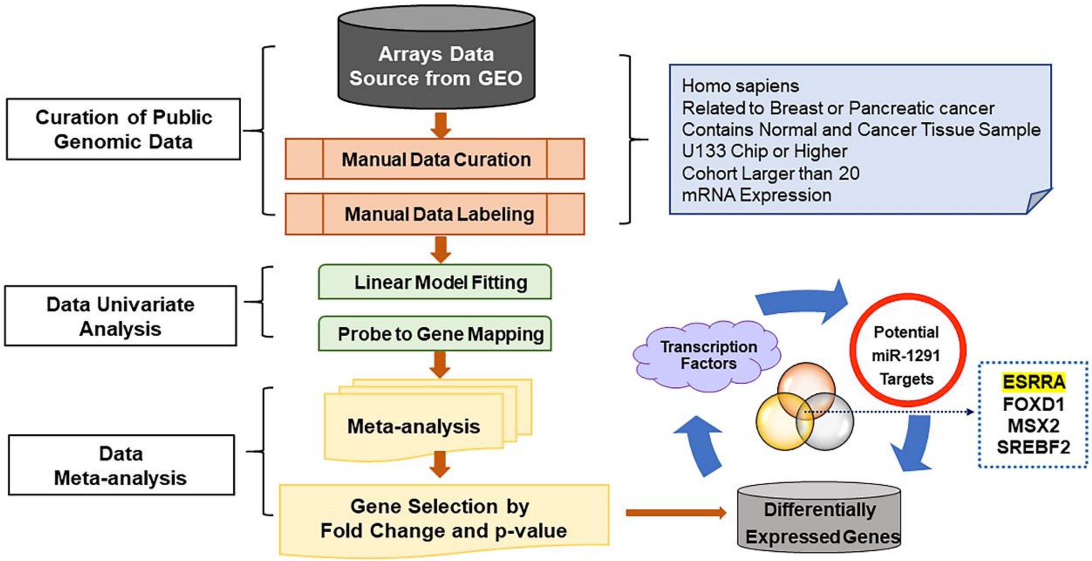

Supplementary Figure S1. The workflow of the Meta-analysis.

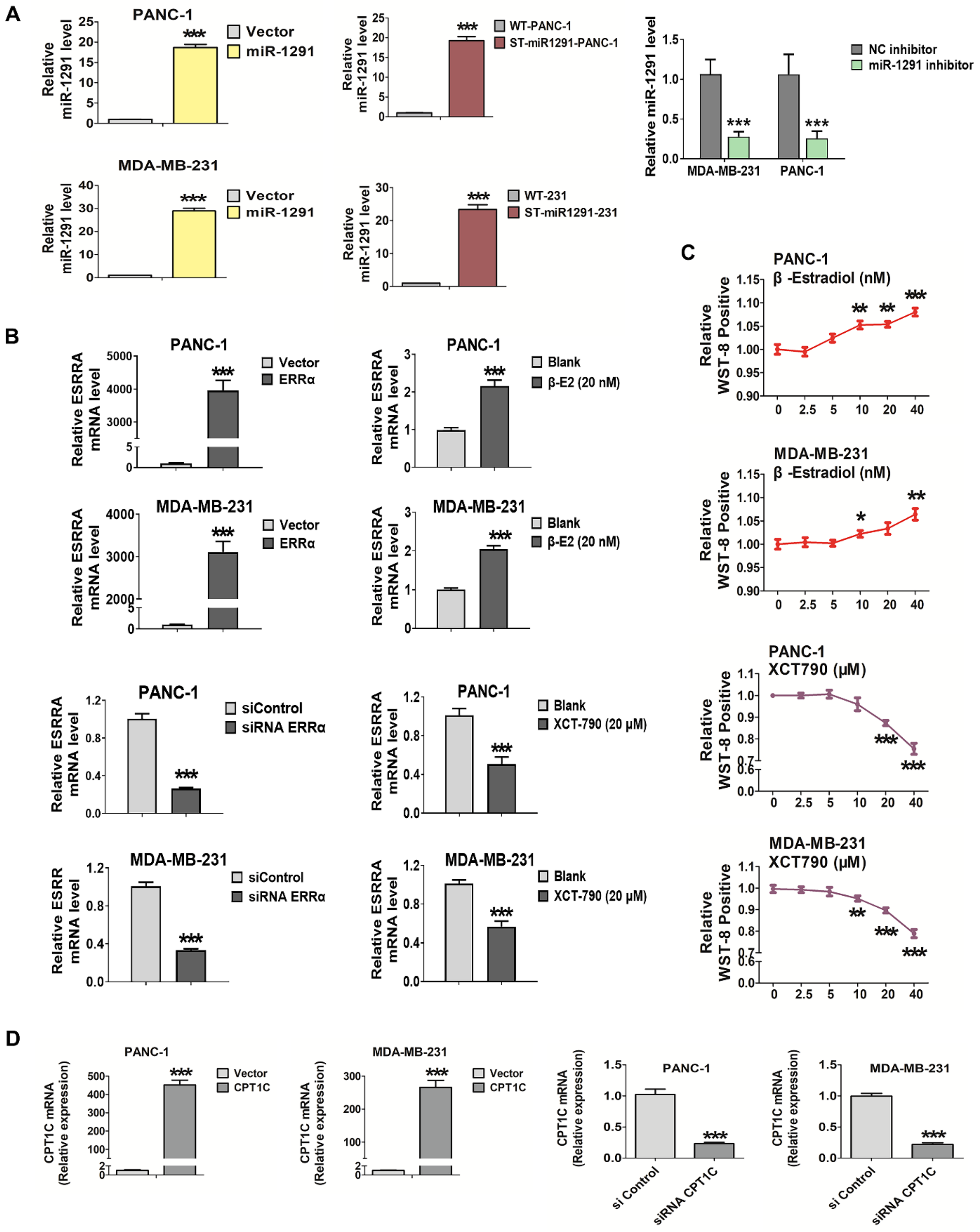

Supplementary Figure S2. (A) RT-qPCR analysis was used to determine the expression of miR-1291 in PANC-1 and MDA-MB-231 cells after transfection with miR-1291 plasmid, miR-1291

inhibitor as well as ST-miR1291 cells. The data are the mean ± SD (n = 6). **(B)** Expression of *ERRα* mRNA in PANC-1 and MDA-MB-231 cells after modulation of ERRα expression and activity with the pENTER-ERRα plasmid and agonist β-E2 (20 nM), respectively, as well as siRNA or chemical inhibitor XCT790 (20 μM). Data are mean ± SD (n = 6). **(C)** WST-8 assays were performed to examine the viability of PANC-1 and MDA-MB-231 cells after the addition of various concentrations of β-E2 or XCT790. Data are mean ± SD (n = 6). **(D)** Expression of *CPT1C* mRNA in PANC-1 and MDAMB-231 cells after modulation of CPT1C expression with the overexpression plasmid and siRNA. Data are mean ± SD (n = 6).

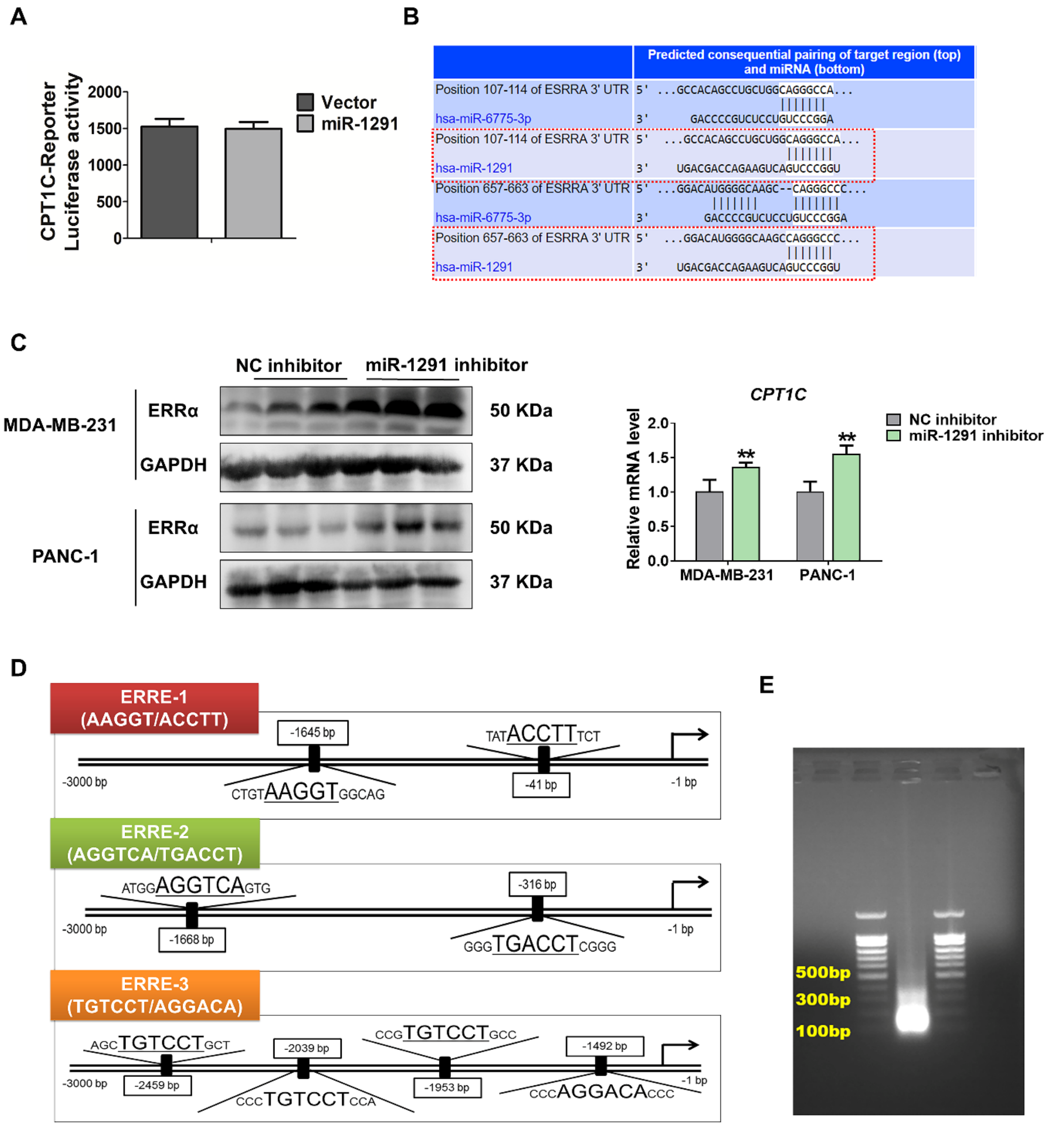

Supplementary Figure S3. (A) *CPT1C* 3'UTR luciferase reporter activity was assessed in HEK-293T cells after transfecting miR-1291. Data are mean ± SD (n = 5). **(B)** Detailed information on the possibility of miR-1291 combined with ERRα. **(C)** ERRα protein expression levels and mRNA levels of *CPT1C* were determined in PANC-1 and MDA-MB-231 cells after transfection with miR-1291 inhibitor. Data are mean ± SD (n = 3 for western-blot, n = 5 for qPCR). **(D)** Eight different ERRE regions predicted in the 3.0 kb CPT1C promoter were identified by a bioinformatics

analysis. The ERRE sequences are denoted as ERRE1, ERRE2, and ERRE3. (**E**) The efficacy of micrococcal nuclease to cutting the DNA fragments in ChIP assay.

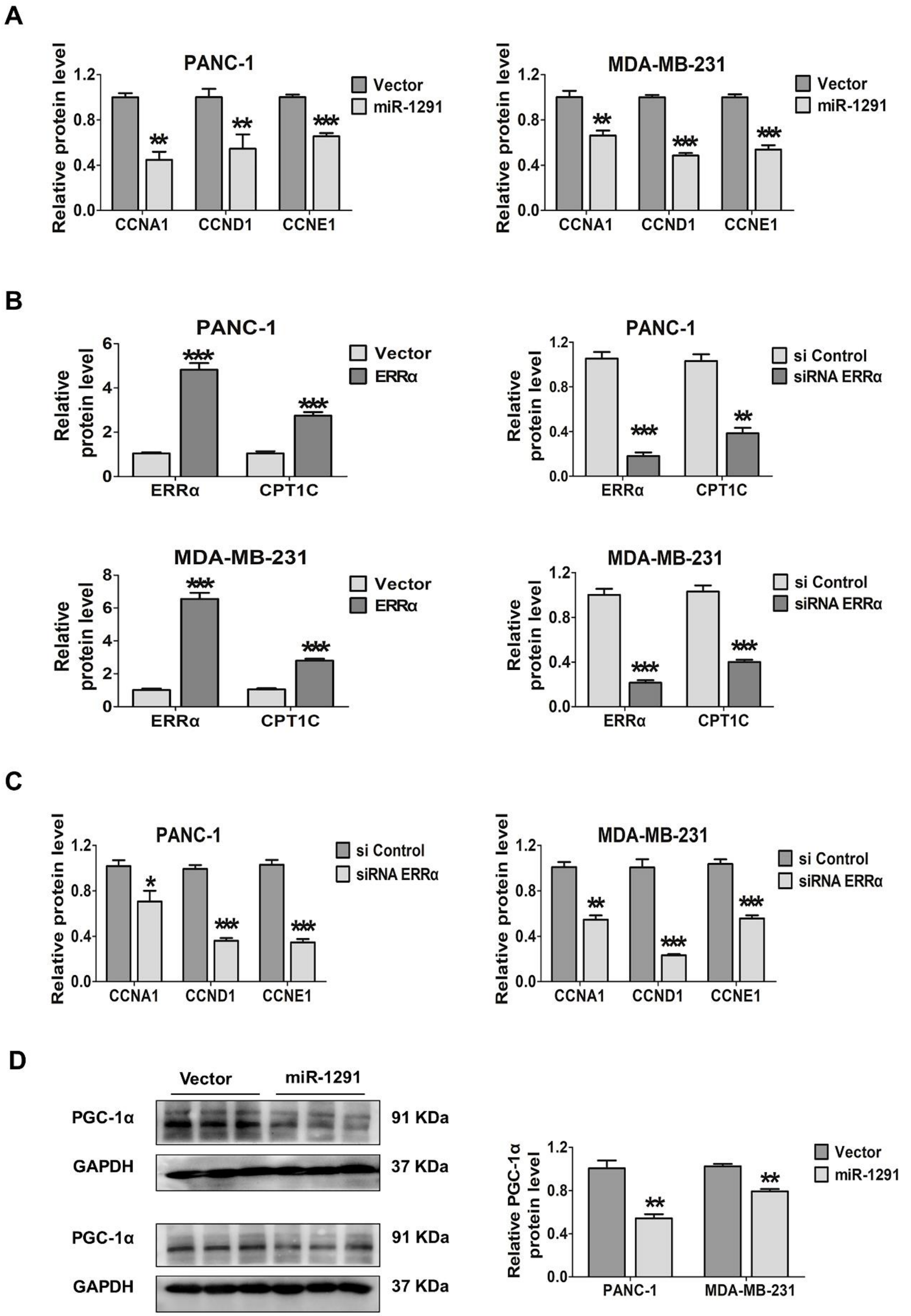
A
PANC-1
Vector
miR-1291
Relative protein level
1.2
0.8
0.4
0.0
**
**
***
CCNA1
CCND1
CCNE1
MDA-MB-231
Vector
miR-1291
Relative protein level
1.2
0.8
0.4
0.0
**
***
***
CCNA1
CCND1
CCNE1
B
PANC-1
Vector
ERRα
Relative protein level
6
4
2
0
***
***
ERRα
CPT1C
PANC-1
si Control
siRNA ERRα
Relative protein level
1.2
0.8
0.4
0.0
***
**
ERRα
CPT1C
MDA-MB-231
Vector
ERRα
Relative protein level
8
6
4
2
0
***
***
ERRα
CPT1C
MDA-MB-231
si Control
siRNA ERRα
Relative protein level
1.2
0.8
0.4
0.0
***
***
ERRα
CPT1C
C
PANC-1
si Control
siRNA ERRα
Relative protein level
1.2
0.8
0.4
0.0
*
***
***
CCNA1
CCND1
CCNE1
MDA-MB-231
si Control
siRNA ERRα
Relative protein level
1.2
0.8
0.4
0.0
**
***
***
CCNA1
CCND1
CCNE1
D
Vector
miR-1291
PGC-1α
91 KDa
GAPDH
37 KDa
PGC-1α
91 KDa
GAPDH
37 KDa
Vector
miR-1291
Relative PGC-1α protein level
1.2
0.8
0.4
0.0
**
**
PANC-1
MDA-MB-231

Supplementary Figure S4. (A) Immunoblot analysis was used to determine cell cycle-related proteins, such as cyclin A/D/E in PANC-1 and MDA-MB-231 cells after transfection with miR-1291. The intensity of protein bands was assayed by Quantity One software and normalized to loading control. Data are mean ± SD (n = 3). **(B)** Western blot analysis was used to measure the protein expression of ERRα and CPT1C in PANC-1 and MDA-MB-231 cells after modulation of ERRα expression. Data are mean ± SD (n = 3). (**C**) The protein levels of cell cycle-related proteins, such as cyclin A/D/E were determined by immunoblot analysis in PANC-1 and MDA-MB-231 cells after transfection with ERRα siRNA. Data are mean ± SD (n = 3). (**D**) Western blot analysis was used to measure the protein expression of PGC-1α in PANC-1 and MDA-MB-231 cells after transfection with miR-1291 plasmid. Data are mean ± SD (n = 3).

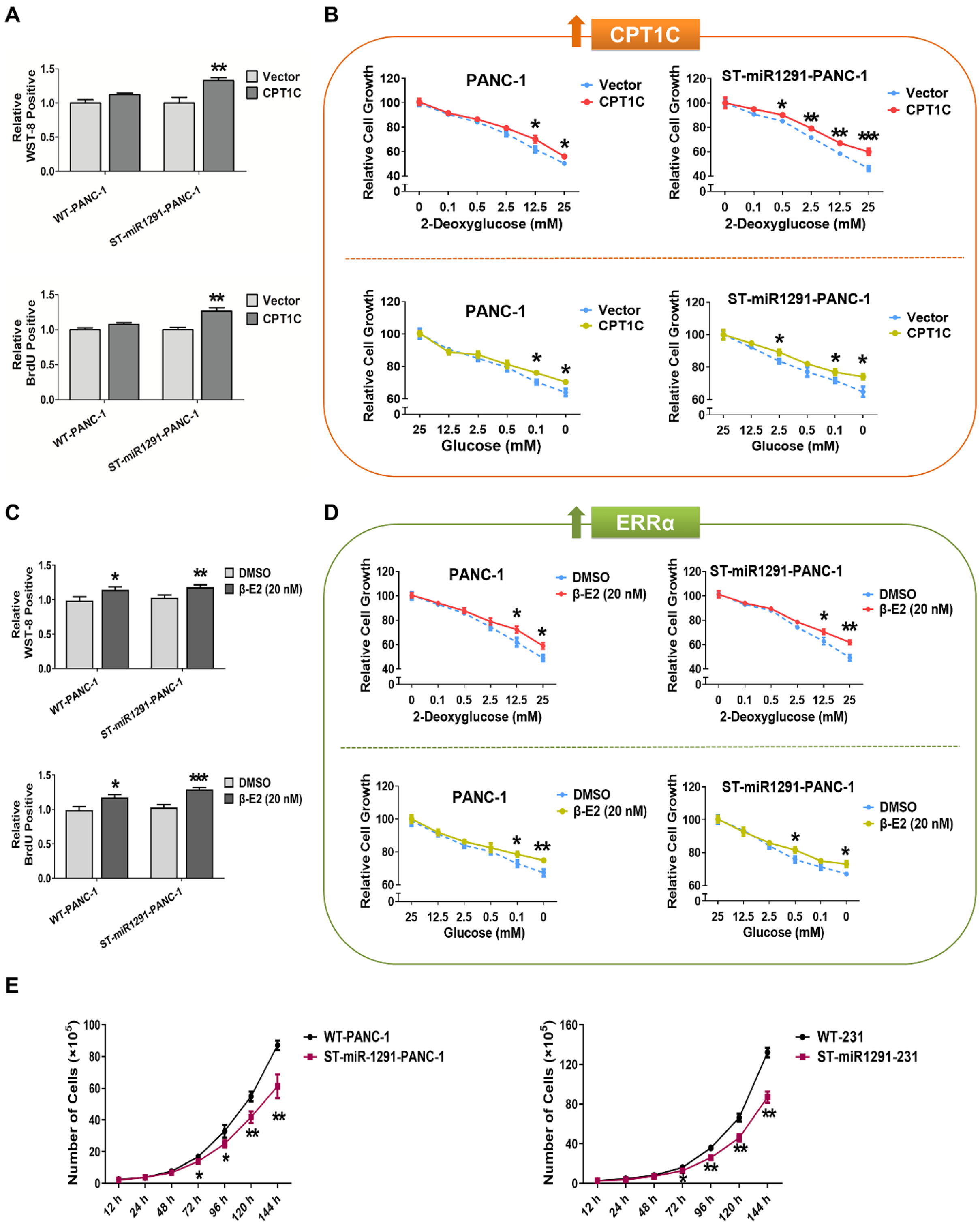

Supplementary Figure S5. (**A**) WST-8 and BrdU assays were performed to examine the effect of high CPT1C expression on the viability and proliferation capacity of WT and ST-miR1291 PANC-

1cells. Data are mean ± SD (n = 5). **(B)** Glycolysis inhibition tests with 2-deoxyglucose and glucose deprivation tests with glucose were performed to measure the impact of overexpression of CPT1C on the anti-metabolic stress ability of WT and ST-miR1291 PANC-1cells. Data are mean ± SD (n = 5). **(C)** WST-8 and BrdU assays were performed to examine the role of ERRα activation on the viability and proliferation capacity of WT and ST-miR1291 PANC-1 cells. Data are mean ± SD (n = 5). **(D)** Glycolysis inhibition tests with 2-deoxyglucose and glucose deprivation tests with glucose were conducted to measure the influence of increased ERRα expression on the anti-metabolic stress ability of WT and ST-miR1291 PANC-1 cells. Data are mean ± SD (n = 5). **(E)** The growth rates of ST-miR-1291 cells and WT cells in different time points. Data are mean ± SD (n = 5).

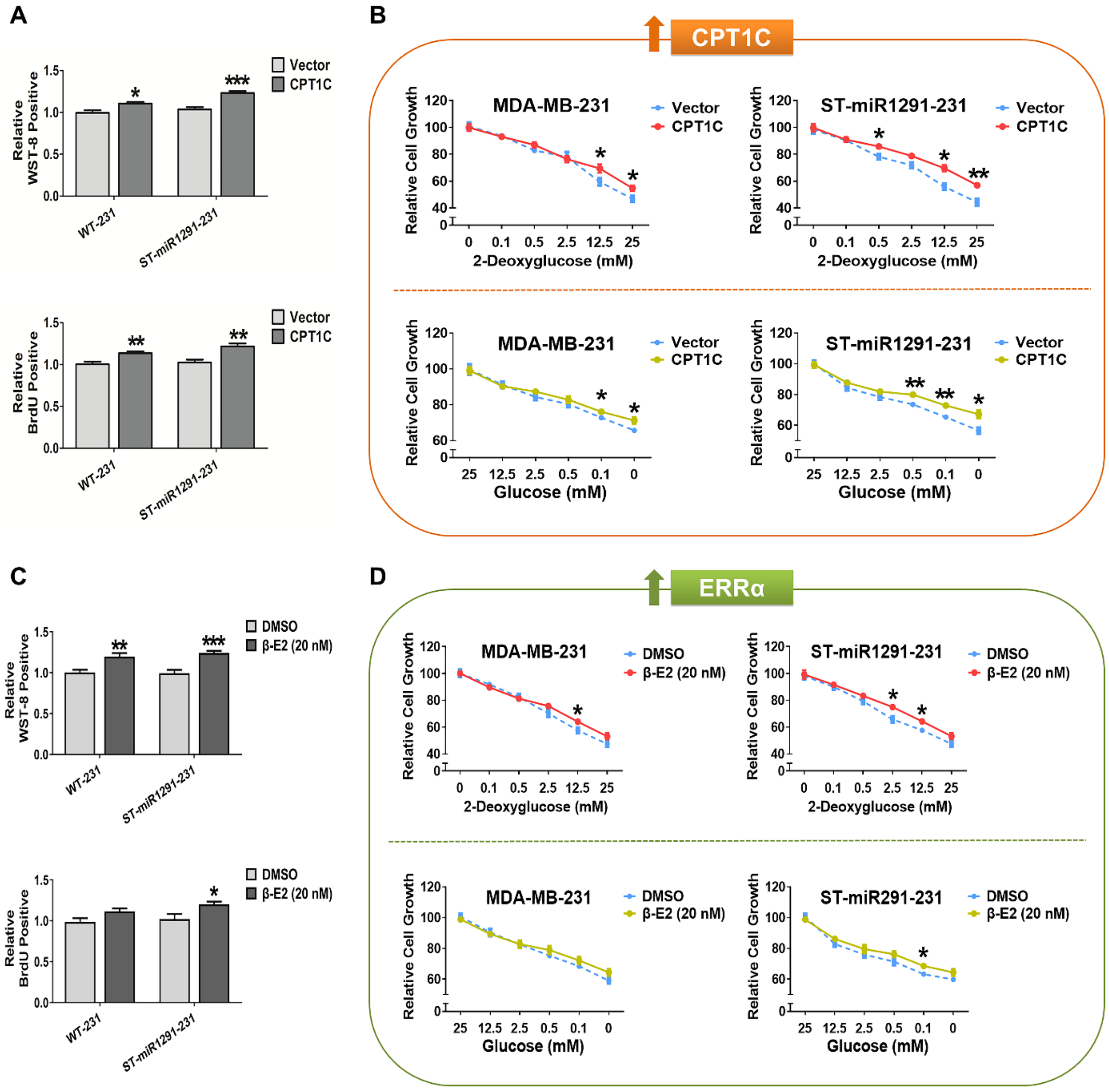

Supplementary Figure S6. (**A**) WST-8 and BrdU assays were performed to examine the effect of high CPT1C expression on the viability and proliferation capacity of WT and ST-miR1291 MDA-MB-231 cells. Data are mean ± SD (*n* = 5). **(B)** Glycolysis inhibition tests with 2-deoxyglucose and glucose deprivation tests with glucose were performed to measure the impact of overexpression of CPT1C on the anti-metabolic stress ability of WT and ST-miR1291 MDA-MB-231 cells. Data are mean ± SD (*n* = 5). **(C)** WST-8 and BrdU assays were performed to examine the role of ERRα activation on the viability and proliferation capacity of WT and ST-miR1291 MDA-MB-231 cells. Data are mean ± SD (*n* = 5). **(D)** Glycolysis inhibition tests with 2-

deoxyglucose and glucose deprivation tests with glucose were conducted to measure the influence of increased ERRα expression on the anti-metabolic stress ability of WT and ST-miR1291 MDA-MB-231 cells. Data are mean ± SD (n = 5).

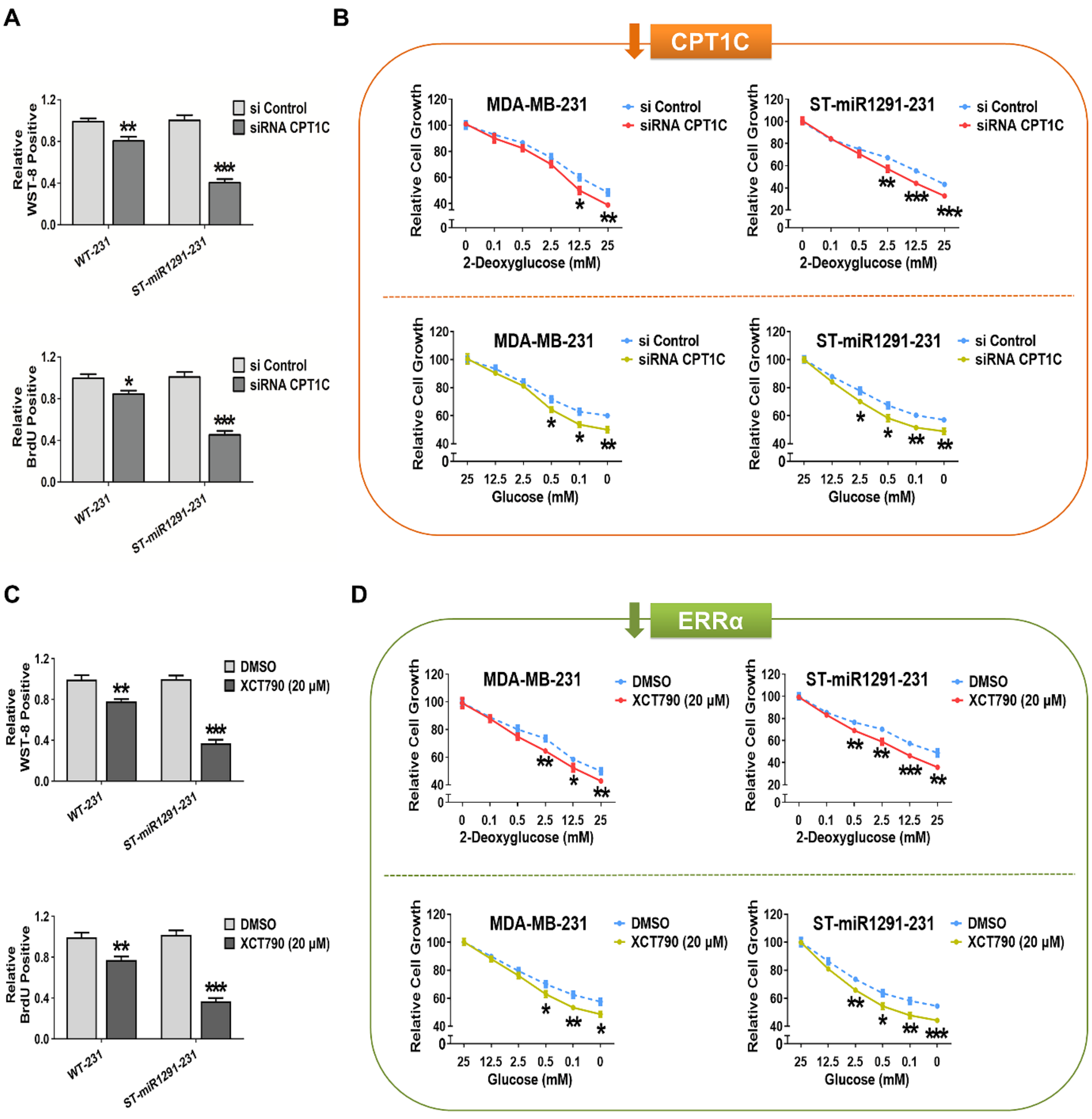

Supplementary Figure S7. (**A**) WST-8 and BrdU assays were performed to examine the effect of low CPT1C expression on the viability and proliferation capacity of WT and ST-miR1291 MDA-MB-231 cells. Data are mean ± SD (*n* = 5). **(B)** Glycolysis inhibition tests with 2-deoxyglucose and glucose deprivation tests with glucose were performed to measure the depletion of CPT1C expression on the anti-metabolic stress ability of WT and ST-miR1291 MDA-MB-231 cells. Data are mean ± SD (*n* = 5). **(C)** WST-8 and BrdU assays were performed to examine the influence of ERRα inhibition on the viability and proliferation capacity of WT

and ST-miR1291 MDA-MB-231 cells. Data are mean ± SD ($n = 5$). **(D)** Glycolysis inhibition tests with 2-deoxyglucose and glucose deprivation tests with glucose were performed to measure the impact of reduction of ERRα expression on the anti-metabolic stress ability of WT and ST-miR1291 MDA-MB-231 cells. Data are mean ± SD ($n = 5$).

Supplemental Tables

Table S1. Sequences of primers for quantitative RT-PCR analysis.

Gene Name	Gene ID	Species Specificity	Sequences of Primers
β-actin	60	Human	forward 5′- CCTTGCACATGCCGGAG-3′<br>reverse 5′-GCACAGAGCCTCGCCTT-3′
ERRα	2101	Human	forward 5′- AGGGTTCCTCGGAGACAGAG-3′<br>reverse 5′- TCACAGGATGCCACACCATAG-3′
CPT1C	126129	Human	forward 5′-GGATGGCACTGAAGAGGAAA-3′<br>reverse 5′-TCCTGGAAAAGGCATCTCTC-3′
PGC-1α	10891	Human	forward 5′- TCTGAGTCTGTATGGAGTGACAT-3′<br>reverse 5′- CCAAGTCGTTCACATCTAGTTCA-3′
GLS	2744	Human	forward 5′-AGGGTCTGTTACCTAGCTTGG-3′<br>reverse 5′- ACGTTCGCAATCCTGTAGATTT-3′
Perlipin-1	5346	Human	forward 5′- TGTGCAATGCCTATGAGAAGG-3′<br>reverse 5′- AGGGCGGGGATCTTTTCCT-3′
STARS	137735	Human	forward 5′- AGCAGTGGGCGAATGAGAAC-3′<br>reverse 5′- GTGATTGGTTTAGGAGCTTGAGG-3′
SPP1	6696	Human	forward 5′- CTCCATTGACTCGAACGACTC-3′<br>reverse 5′- CAGGTCTGCGAAACTTCTTAGAT-3′
EGF	1950	Human	forward 5′- TGGATGTGCTTGATAAGCGG-3′<br>reverse 5′- ACCATGTCCTTTCCAGTGTGT-3′
TFF1	7031	Human	forward 5′- CCCCGTGAAAGACAGAATTGT-3′<br>reverse 5′- GGTGTCGTCGAAACAGCAG-3′
NRF-1	4899	Human	forward 5′- AGGAACACGGAGTGACCCAA-3′<br>reverse 5′- TATGCTCGGTGTAAGTAGCCA-3′
TFAM	7019	Human	forward 5′- ATGGCGTTTCTCCGAAGCAT-3′<br>reverse 5′- TCCGCCCTATAAGCATCTTGA-3′
CYBA	1535	Human	forward 5′- CCCAGTGGTACTTTGGTGCC-3′<br>reverse 5′- GCGGTCATGTACTTCTGTCCC-3′

Table S2. Sequences of primers for miRNA quantitative RT-PCR analysis.

Name	Sequences of Primers
MiRNA Stem-loop Primer	5′-GTCGTATCCAGTGCAGGGTCCGAGGT ATTCGCACTGGATACGACACTGCT-3′
U6-F	forward 5′-CTCGCTTCGGCAGCACA-3′
U6-R	reverse 5′- AACGCTTCACGAATTTGCGT-3′
miR-1291-F	forward 5′- CGTGGCCCTGACTGAAGACC -3′
miR-1291-F	reverse 5′- AGTGCAGGGTCCGAGGTATT -3′

Table S3. Sequences of primers for ChIP-qPCR analysis.

Name	Sequences of Primers
ERR-CPT1C-chip-1F	forward 5′- GAATGGCTTGGGGCTTAGGG-3′
ERR-CPT1C-chip-1R	reverse 5′-AGTTGCACTGAAGCAGGTGTAGC-3′
ERR-CPT1C-chip-2F	forward 5′- TTCTGTGGATCTGCGTCTCCC-3′
ERR-CPT1C-chip-2R	reverse 5′- TCGAGTGTTGGGGGGAGG-3′
ERR-CPT1C-chip-3F	forward 5′-TGGGCGCCGCCGGTGGCG-3′
ERR-CPT1C-chip-3R	reverse 5′-AGTTAGGGGAGAAGAAATGTGGAGTAGAAGC-3′
ERR-CPT1C-chip-4F	forward 5′- GGGACCAGGCTGGGCGAA-3′
ERR-CPT1C-chip-4R	reverse 5′- ACTTCCGTGAGGGAGAAGCAG-3′

Table S4. Microarray GSE data summarization

CANCER	GSE Number	GPL	PMID	YEAR	Samples Number	
					NORMAL	CANCER
BREAST CANCER	GSE10780	GPL570	19266279	2009	101	42
	GSE10810	GPL570	20029976	2009	21	37
	GSE15852	GPL96	20097481	2009	43	43
	GSE20437	GPL96	20197764	2010	24	18
	GSE22544	GPL570	20799942	2010	4	16
	GSE25407	GPL570	21118987	2010	5	5
	GSE29431	GPL570	#N/A	2011	12	54
	GSE42568	GPL570	23740839	2013	17	104
	GSE5764	GPL570	17389037	2007	20	10
	GSE61304	GPL570	#N/A	2015	4	58
	GSE7904	GPL570	16473279	2007	19	43
	GSE9574	GPL96	18058819	2007	15	14
PANCREATIC CANCER	GSE15471	GPL570	19260470	2009	42	36
	GSE18670	GPL570	23157946	2012	6	18
	GSE19650	GPL570	20955708	2010	7	15
	GSE22780	GPL570	#N/A	2011	8	8
	GSE27890	GPL570	#N/A	2014	4	6
	GSE46234	GPL570	#N/A	2017	4	4
